# Interstitial boron-triggered electron-deficient Os aerogels for enhanced pH-universal hydrogen evolution

**DOI:** 10.1038/s41467-022-28805-8

**Published:** 2022-03-03

**Authors:** Yinghao Li, Chun-Kuo Peng, Huimin Hu, San-Yuan Chen, Jin-Ho Choi, Yan-Gu Lin, Jong-Min Lee

**Affiliations:** 1 School of Chemical and Biomedical Engineering, Nanyang Technological University, 62 Nanyang Drive, Singapore, 637459 Singapore; 2 Department of Material Science and Engineering, National Yang Ming Chiao Tung University, Hsinchu, 30010 Taiwan; 3 Soochow Institute for Energy and Materials Innovations & Key Laboratory of Advanced Carbon Materials and Wearable Energy Technologies of Jiangsu Province, College of Energy, Soochow University, Suzhou, 215006 China; 4 Scientific Research Division, National Synchrotron Radiation Research Center, Hsinchu, 30076 Taiwan

**Keywords:** Electrocatalysis, Nanoscale materials, Catalytic mechanisms

## Abstract

Developing high-performance electrocatalysts for hydrogen evolution reaction (HER) is crucial for sustainable hydrogen production, yet still challenging. Here, we report boron-modulated osmium (B-Os) aerogels with rich defects and ultra-fine diameter as a pH-universal HER electrocatalyst. The catalyst shows the small overpotentials of 12, 19, and 33 mV at a current density of 10 mA cm^−2^ in acidic, alkaline, and neutral electrolytes, respectively, as well as excellent stability, surpassing commercial Pt/C. Operando X-ray absorption spectroscopy shows that interventional interstitial B atoms can optimize the electron structure of B-Os aerogels and stabilize Os as active sites in an electron-deficient state under realistic working conditions, and simultaneously reveals the HER catalytic mechanisms of B-Os aerogels in pH-universal electrolytes. The density functional theory calculations also indicate introducing B atoms can tailor the electronic structure of Os, resulting in the reduced water dissociation energy and the improved adsorption/desorption behavior of hydrogen, which synergistically accelerate HER.

## Abstract (web summary)

While noble metals can be active electrocatalysts for producing renewable H_2_, there are relatively few works examining osmium materials. Here, the authors prepare boron-doped osmium aerogels for H_2_ evolution electrocatalysis plus examine the mechanism using computational and in situ characterization.

## Introduction

Hydrogen, a carbon-free renewable energy source with the highest gravimetric energy density, is widely regarded as a useful alternative for conventional fossil fuels to solve the problems of fast-consuming fossil fuels, ever-increasing terawatt energy demand and severe environmental situation^1–3^. Among all hydrogen production methods, electrocatalytic water splitting driven by renewable electricity is an environmentally compatible, cheap, and feed-rich technology to produce high-purity hydrogen, which has attracted ongoing interests^4,5^. However, platinum (Pt) is still the state-of-the-art electrocatalyst for hydrogen evolution reaction (HER), especially in acid electrolytes, on account of its fast kinetic mechanism and optimal bonding strength with hydrogen^6,7^. Nonetheless, apart from the high cost and low reserves, the poor stability induced by leaching in corrosive solution and unexpected aggregation of Pt nanoparticles (NPs), and the suboptimum activity under alkaline and neutral conditions resulted from the slow dynamics of water dissolution step (H_2_O→H*+OH^−^), have severely hampered its large-scale applications^8–10^. With regard to practical water-splitting applications, electrocatalysts need to perform well in the wide pH range including acidic electrolytes for proton-exchange membrane-based electrolysis cells, neutral electrolytes for microbial electrolysis cells and alkaline electrolytes for water-alkali and chlor-alkali electrolyze technology^11,12^. Consequently, designing and developing cost-effective Pt-free electrocatalysts with high activity and durability in pH-universal electrolytes for HER is of the essence yet challenging.

Noble-metal aerogels (NMAs), the utmost important category of noble-metal foams, have excited considerable attention upon their debut in 2009 owing to simple preparation strategies, flexible composition control, and the perfect inheritance of foam characteristics (high inner surface area, 3D self-supporting network structure, and large porosity) as well as noble-metal peculiarity (excellent electrical conductivity, remarkable catalytic activity, and unique plasmonic features)^13–15^. These distinct physicochemical features endow NMAs with numerous active sites, excellent structural stability, and fast proton/electron transport, which make them widely used in electrocatalysis^16,17^, sensing^18,19^, and surface-enhanced Raman scattering^20,21^. For example, Du et al. reported that the Au-Rh aerogels and Au-Pt aerogels exhibited excellent electrocatalytic performance for pH-universal HER and oxygen reduction reaction, respectively, surpassing commercial Pt/C and many other NP-based catalysts^22^. Zhu et al. reported that the PdCu bimetallic aerogels showed enhanced catalytic activity and durability for ethanol oxidation in acidic solutions compared to commercial Pd/C^23^. Nevertheless, in contrast to Au^24^, Ag^25^, Pd^13^, Pt^26^ and their alloy aerogels^27,28^ that have been extensively studied, the preparation and application of monometallic Ir, Rh, Ru, Os and their alloy aerogels are rarely reported^15,29^. Among various noble metals, Os, as a member of Pt group metals, has received less attention, especially in the field of electrocatalysis. More importantly, the price of Os (400 USD per ounce) is much less than that of Pt (1094 USD per ounce), which makes it a prospective substitute for electrocatalysis. Some studies showed that preparing NMAs with ultra-fine diameters to maximize atomic utilization is an effective and challenging strategy to improve the performance of the catalyst^23^. Meanwhile, relative to the monometallic aerogels, metal-heteroatom alloying is regarded as one of the most useful pathways to further improve the catalytic performance by redistributing the electron density of catalysts and optimizing the adsorption energy of the reactant species on the catalyst surface^30–34^. For instance, Lin et al. synthesized Pd-Ni-P ternary NPs with a diameter of about 5 nm via a two-step solvothermal method, which shows excellent mass activity (4.95 A mg^−1^_Pd_) for ethanol electrooxidation in an alkaline electrolyte with a P-induced optimal reactive-intermediate pathway^35^. Zhou et al. reported that B-doped copper catalysts with 1.7% boron content demonstrated superior activity and stability for electrocatalytic CO_2_ reduction reaction (CO_2_RR) due to the average valence of copper regulated by B atoms^33^. In addition, the in-depth exploration of the real catalytic process and the identification of active-site structure are rarely available due to the structural remodeling, elemental valence changes and transformation of exposed active sites of electrocatalysts under realistic reaction conditions, probably resulting from additional electric potential, reaction temperature, or corrosive electrolyte^36,37^. In situ/operando characterizations, using X-ray absorption spectroscopy (XAS), offer an appropriate way to overcome these limitations, which can probe the changes in the dynamic electronic structure of catalysts and adsorbed intermediates during an actual reaction^38–40^. Therefore, from the perspective of basic research, the development and utilization of Os and the understanding of its electrocatalytic reaction mechanism at the atomic level are critical but utmost challenging for the rational design of future electrocatalysts with high performance.

Herein, we report a trace boron-doped Os aerogel (B-Os aerogel) with an ultra-fine diameter (~1.7 nm) and abundant defects via a simple one-step sol–gel method, using NaBH_4_ as reductant and dopant. Benefitting from the distinct physicochemical features and optimized electronic structure induced by the intervening boron atoms, the as-prepared B-Os aerogel demonstrates high activity and superior stability in all pH ranges, especially with the high mass activity of 1175.36 A g_Os_^−1^ and price activity of 83.33 A dollar^−1^ at an overpotential of 100 mV in 1.0 M KOH solution, outperforming most of reported advanced HER electrocatalysts and other noble-metal aerogels even including Pt. X-ray absorption near edge structure (XANES) spectra and density functional theory (DFT) calculations identify the Os-B configuration as the most plausible structure of the active site, which possesses low energy barriers of water dissociation and adequate hydrogen binding energies to accelerate HER over a wide pH range. More importantly, operando XAS indicates that B can stabilize osmium in an electron-deficient state, contributing to the excellent activity and stability, and also reveals the mechanisms of B-Os aerogels in varied electrolyte environments, which is valuable for the rational design of future electrocatalysts with high performance.

## Results

### Synthesis and characterization of B-Os aerogels

**Fig. 1:**
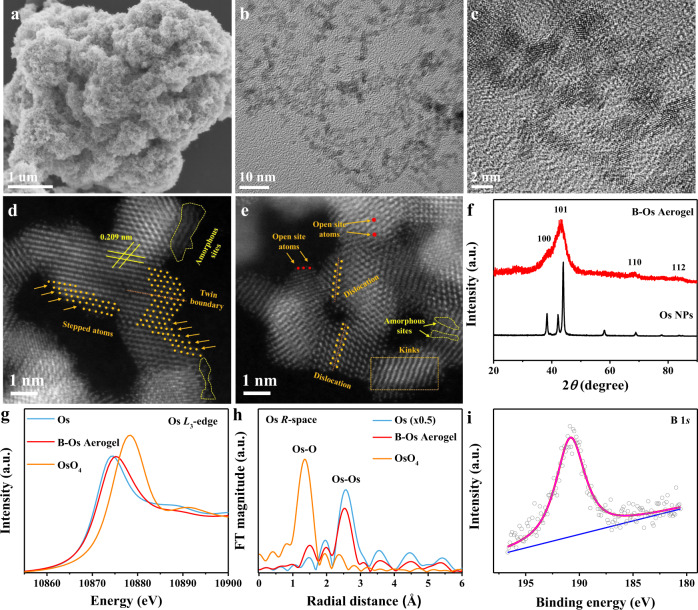
Morphological and structure analysis of B-Os aerogels. **a** SEM; **b**, **c** HRTEM; **d**, **e** ACTEM images of B-Os aerogels. **f** XRD patterns of B-Os aerogels and Os NPs. **g** Os *L*_3_-edge XANES spectrum of B-Os aerogels with reference metallic Os and OsO_4_. **h** Fourier transformation of the EXAFS spectra of B-Os aerogels with reference metallic Os and OsO_4_. **i** Boron XPS spectrum.

The B-Os aerogels were synthesized from OsCl_3_ aqueous solutions by a NaBH_4_-induced gelation process, where NaBH_4_ was served as both reducing agent and boron dopant. During the gelation process, a great deal of hydrogen produced by the hydrolysis of NaBH_4_ acts as gas templates to guide the generation of the porous nanostructure. After washing and drying, monolithic aerogels were obtained (Supplementary Fig. 1). Scanning electron microscopy (SEM) and transmission electron microscopy (TEM) images (Fig. 1a, b) clearly indicate that as-prepared B-Os aerogels possess a 3D porous architecture constructed by ultrathin interconnected nanowires. The fine structure of these nanowire networks was further analyzed with high-resolution TEM. As shown in Fig. 1c and Supplementary Fig. 2, the network morphology with abundant pores and bifurcations consists of ultra-fine nanowires (average diameter ~1.7 nm) randomly interlinked at different angles, which is conducive to the transport of electrolyte and the exposure of enriched active sites during electrocatalysis. Besides, the surface area and porosity features of the B-Os aerogel were also analyzed with the N_2_ physisorption isotherm; its surface area is 86.52 m^2^ g^−1^ according to the Brunauer–Emmett–Teller (BET) model (Supplementary Fig. 3a); the pore diameter in Supplementary Fig. 3b is widely distributed from micropores (<2 nm) to mesopores (2–30 nm). The aberration-corrected scanning TEM images in Fig. 1d, e indicate that rich defects including open sites, stacking faults, amorphous sites, low-coordinated stepped atoms, kinks and twin boundaries were clearly observed on the basal surface and edges of the B-Os aerogel. These abundant defects are regarded as highly active sites, which can optimize the electronic structure of catalysts via destroying the original charge distribution, remodeling new charge balance, and creating local charge aggregation, thereby boosting their electrocatalytic activity^41–43^. In addition, the lattice fringe demonstrated in Fig. 1d is 0.209 nm, which is designated as the (101) plane of face-centered cubic (fcc) Os, and the basal plane was dominantly hosted by the (101) facets. Interestingly, the B-Os aerogels show a larger interplanar spacing than that of pure Os (0.207 nm). The expansion of the Os-Os lattice can be attributed to the intervention of B atoms in the lattice gaps, which was further confirmed by the negative shift of the X-ray diffraction (XRD) peaks, relative to those of pure Os NPs (Fig. 1f)^44^. Os and B are therefore confirmed to form interstitial nanocrystals, similar to previous reports^45,46^.

The local electronic structure of B-Os aerogels was first investigated with XAS. Figure 1g demonstrates the Os *L*_3_-edge XANES spectra of B-Os aerogels with reference metallic Os and OsO_4_. The rising edge of the signal from B-Os aerogels shows a positive shift compared with that from metallic Os due to the improved valence oxidation state after the intervention of B into the lattice of Os. This phenomenon is ascribed to a shift of electron density from metallic Os to local B atoms with high electronegativity^39^. The chemical composition and surface electronic states of B-Os aerogels were further explored by X-ray photoelectron spectroscopy (XPS). The peaks at 51.1 and 53.9 eV in Supplementary Fig. 4 are assigned to the 4*f*_7/2_ and 4*f*_5/2_ of Os^0^, respectively, while those at 52.0 and 55.3 eV are designated as the 4*f*_7/2_ and 4*f*_5/2_ levels of Os^4+^. Obviously, compared with that in pure Os (4*f*_7/2_, 50.7 eV), the 4*f*_7/2_ state of Os in the B-Os aerogel was elevated by 0.4 eV, indicating the strong electron transfer from Os to B atoms and the downshifted d-band center of surface Os atoms, which is in accordance with XANES analysis, similar to the previous B-Pd^34^, B-Pt^47^ and B-Cu systems^33^. The surface Os(ΙV) component for B-Os aerogels might result from the aging exposure to the air. Moreover, the presence of B in the B-Os aerogel was confirmed in Fig. 1i and other elements, like Cl and Na, were not found (Supplementary Fig. 5), proving that B is only doped into Os during the sample preparation. About 0.67% of B in the B-Os aerogel was further detected using inductively coupled plasma optical emission spectroscopy (ICP-OES). Furthermore, the local atomic structure of B-Os aerogels was investigated with extended X-ray absorption fine-structure (EXAFS) spectra. Os *L*_3_-edge k^3^χ(k) oscillation spectra (Supplementary Fig. 6) showed a difference between the B-Os aerogel and the metallic Os reference, implying a different component in Os for the B-Os aerogel. In the Os *L*_3_-edge k^3^-weighted EXAFS spectra, two peaks at 1.53 and 2.53 Å were ascribed to Os-B and Os-Os (Fig. 1h). The positive shift peak at 1.53 Å in the B-Os aerogel clarified the effect of B substitution compared with the Os-O peak at 1.35 Å in Os-O_4_.

To understand the effect of the synthetic conditions, several control experiments were performed. When OsCl_3_ solution at an extremely low concentration (1.0 mM) was used, there was no porous network structure formed regardless of the amount of NaBH_4_ added (Supplementary Fig. 7a). When OsCl_3_ solution (10 mM) was used, traces of aerogels began to appear (Supplementary Fig. 7b). The optimized concentration of OsCl_3_ (50 mM) highly supports the synthesis of the high-quality B-Os aerogels (Supplementary Fig. 7c). Further increasing the OsCl_3_ concentration to 0.1 M generated irregular NPs due to the agglomeration of numerous reduced NPs (Supplementary Fig. 7d). These results indicate that OsCl_3_ concentration is of great significance for the formation of B-Os aerogels. Furthermore, the role of the ratio of OsCl_3_ to NaBH_4_ was explored during the preparation of B-Os aerogels with varied ratios of OsCl_3_ (50 mM) to NaBH_4_ (50 mM). When the molar ratio of OsCl_3_ to NaBH_4_ was 2:1, large ligament B-Os networks formed owing to the slow reduction rate (Supplementary Fig. 8a). Nevertheless, the mixture of OsCl_3_ and NaBH_4_ at an equal ratio led to small NPs (Supplementary Fig. 8b), whereas the ratio of OsCl_3_ to NaBH_4_ greater than 1 supported the formation of aerogels (Supplementary Fig. 8c, d), indicating that the ratio of the precursor to the reducing agent plays a key role in adjusting the morphology of the B-Os aerogel. The selection of NaBH_4_ as a reducing agent is also indispensable for the formation of B-Os aerogel. When we use hydrazine hydrate, sodium sulfite or ascorbic acid instead of NaBH_4_ as a reducing agent, under the same synthesis conditions, only low-quality osmium oxide aerogels or irregular NPs can be obtained due to the lack of gas template and relatively weak reduction ability (Supplementary Fig. 9a–f). The mixture of OsCl_3_ and NaBH_4_ at the optimal ratio and concentration first produced a large number of nucleation sites (reduced Os nanoclusters). Simultaneously, the BH_4_^–^ ions were decomposed on the surface of Os NPs to generate B atoms, followed by the diffusion of these formed B atoms into the Os-Os lattice. Subsequently, the rapid fusion and growth of these nanoclusters, oriented attachment of short chains as well as the ripping of the interconnected nanowires resulted in the formation of high-quality B-Os interstitial alloy aerogels.

### Electrocatalytic performance toward HER

**Fig. 2:**
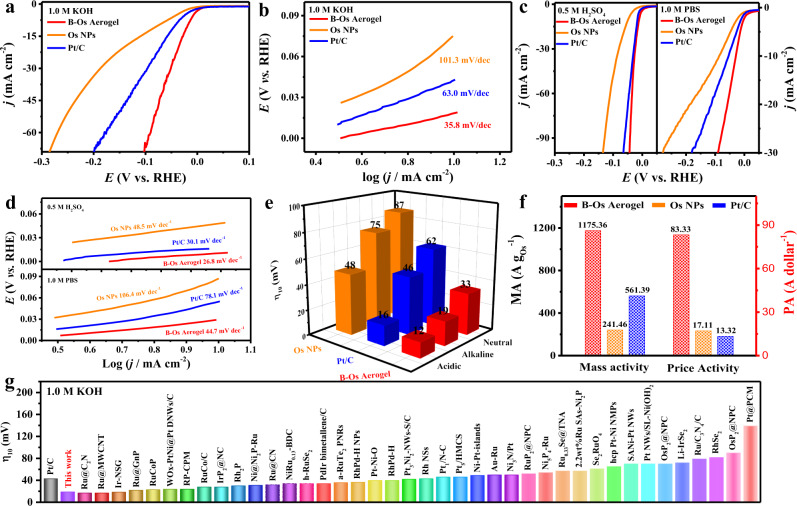
Electrochemical HER performance measurements. **a**, **b** LSV curves and corresponding Tafel plots of B-Os aerogels, Os NPs and Pt/C in 1.0 M KOH solution. **c**, **d** LSV curves and corresponding Tafel plots of B-Os aerogels, Os NPs and Pt/C in 0.5 M H_2_SO_4_ and 1.0 M PBS solutions, respectively. **e** Overpotential at 10 mA cm^−2^ of B-Os aerogels, Os NPs and Pt/C in 1.0 M KOH, 0.5 M H_2_SO_4_ and 1.0 M PBS solutions, respectively. **f** Mass activity (MA) and price activity (PA) of B-Os aerogels, Os NPs and Pt/C at an overpotential of 100 mV in 1.0 M KOH solution. **g** Comparison of the overpotentials at 10 mA cm^−2^ (η_10_) with recently reported HER catalysts in 1.0 M KOH solution.

Due to the unavoidable effect of proton or hydroxide concentration on the electrocatalytic activity during electrolysis, a promising catalyst is expected to perform efficiently in a universal pH range. Consequently, the HER performance of B-Os aerogels was assessed in KOH (1.0 M, pH 14), H_2_SO_4_ (0.5 M, pH 0), and phosphate buffer (PBS, 1.0 M, pH 7) solutions using a three-electrode configuration at ambient temperature. For comparison, Os NPs and commercial Pt/C were measured as benchmarks under identical conditions. As shown in Fig. 2a, the B-Os aerogel demonstrated outstanding HER performance, showing a near-zero onset potential in the alkaline solution. Impressively, it required an overpotential of only 19 mV to achieve a cathodic current density of 10 mA cm^−2^, which is substantially lower than those of Os NPs (75 mV) and commercial Pt/C (46 mV) (Fig. 2e). Furthermore, B-Os aerogels produced a high current density (100 mA cm^−2^) at a low overpotential of 137 mV in the alkaline electrolyte, which is much better than Os NPs and Pt/C. Remarkably, the Tafel slope of B-Os aerogels (35.8 mV dec^−1^) is less than those of Os NPs (101.3 mV dec^−1^) and commercial Pt/C (63.0 mV dec^−1^), indicating that the B-Os aerogel has the faster HER kinetic essence through the Volmer–Heyrovsky mechanism and the electrochemical desorption step on the catalyst surface is the rate-limiting step (Fig. 2b)^48,49^. The exchange current density of B-Os aerogels (3.2 mA cm^−2^), calculated by extrapolating the Tafel plots, was 2.3 and 1.5 times greater than those of Os NPs (1.4 mA cm^−2^) and Pt/C (2.1 mA cm^−2^), respectively, which also confirmed that the B-Os aerogel possesses the better HER kinetics and improved intrinsic performance in the alkaline solution (Supplementary Fig. 10). In addition, the B-Os aerogel also exhibited superior HER activities with overpotentials of 12 and 33 mV to reach a current density of 10 mA cm^−2^ in 0.5 M H_2_SO_4_ and 1 M PBS, respectively, than Os NPs and Pt/C in Fig. 2c, e. The Tafel slopes of B-Os aerogels in 0.5 M H_2_SO_4_ and 1 M PBS were approximately 26.8 and 44.7 mV dec^−1^, indicating that the B-Os aerogel has the more beneficial HER kinetics than Os NPs and Pt/C (Fig. 2d)^50–52^. Compared with those in the acidic condition, the higher Tafel slopes in the alkaline and neutral solution are largely owing to the slow kinetic process of water decomposition before the adsorption of the generated hydrogen atoms on the catalytic active sites^53^. Similarly, the higher exchange current density of the B-Os aerogel in the acidic medium (3.6 mA cm^−2^) and neutral medium (2.3 mA cm^−2^) also proved that it has the better HER electrocatalytic property (Supplementary Figs. 11 and 12). For the sake of a fair evaluation of electrocatalytic activity, the mass and price activity of B-Os aerogels were calculated based on the difference in the price of Os and Pt (Supplementary Table 1), which are important criteria to evaluate the potential for practical applications of catalysts. As shown in Fig. 2f and Supplementary Fig. 13, compared to Os NPs and Pt/C, B-Os aerogels demonstrated the highest mass activity at an overpotential of 100 mV in 1.0 M KOH solution (1175.36 A g_Os_^−1^), at an overpotential of 50 mV in 0.5 M H_2_SO_4_ solution (2505.37 A g_Os_^−1^) and at an overpotential of 100 mV in 1.0 M PBS solution (580.38 A g_Os_^−1^), respectively. Specifically, at the overpotential of 100 mV, the price activity of B-Os aerogels was as high as 83.33 A dollar^−1^ in the alkaline electrolyte, which was 4.9 and 6.3 times higher than those of the Os NPs (17.11 A dollar^−1^) and Pt/C (13.32 A dollar^−1^), respectively (Fig. 2f). Moreover, the B-Os aerogel also displayed much better price activities than Os NPs and Pt/C at an overpotential of 50 mV in the acidic electrolyte (177.63 A dollar^−1^) and at an overpotential of 100 mV in the neutral electrolyte (41.15 A dollar^−1^) (Supplementary Fig. 13). The remarkable electrocatalytic activities of the B-Os aerogel overwhelmingly exceeded most recently reported noble-metal-based HER electrocatalysts over a wide pH range (Fig. 2g and Supplementary Tables 2–4).

**Fig. 3:**
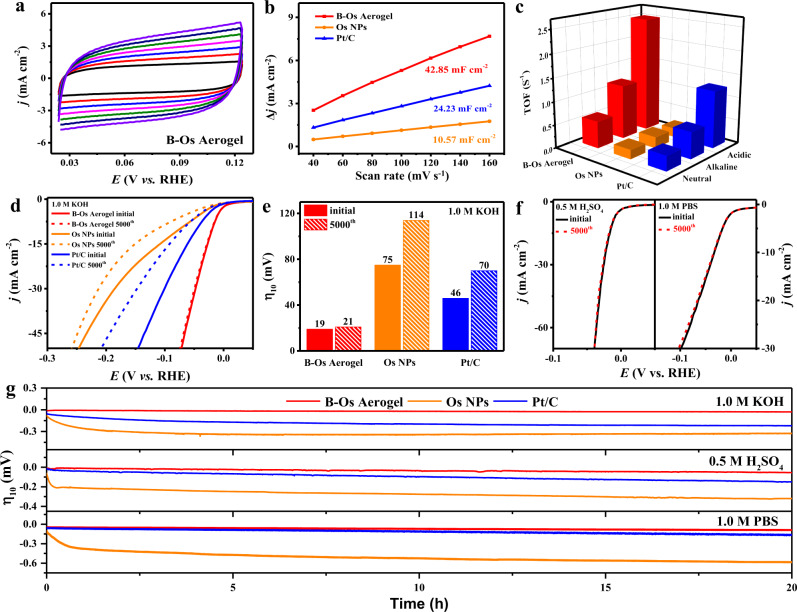
Comparison of electrochemical HER parameters. **a**, **b** Cyclic voltammetry (CV) plots of B-Os aerogels with varying scan rates from 40 to 160 mV s^−1^ and *C*_dl_ measurement of B-Os aerogels, Os NPs and Pt/C in 1.0 M KOH solution. **c** TOF values of B-Os aerogels, Os NPs and Pt/C in 1.0 M KOH, 0.5 M H_2_SO_4_ and 1.0 M PBS solutions, respectively. **d**, **e** iR-corrected polarization curves and corresponding overpotential changes at 10 mA cm^−2^ of B-Os aerogels, Os NPs and Pt/C were recorded before and after 5000 CV potential cycles in 1.0 M KOH solution. **f** iR-corrected polarization curves of B-Os aerogels were recorded before and after 5000 CV potential cycles in 0.5 M H_2_SO_4_ and 1.0 M PBS solutions, respectively. **g** The chronopotentiometry of B-Os aerogels, Os NPs and Pt/C in 1.0 M KOH, 0.5 M H_2_SO_4_ and 1.0 M PBS solutions, respectively.

In order to clarify the source of the increased electrocatalytic activity of the B-Os aerogel, we calculated the electrochemical double-layer capacitance (*C*_dl_) by undertaking cyclic voltammetry (CV) at different scan rates. As shown in Fig. 3a, b and Supplementary Figs. 14–16, the B-Os aerogel had the *C*_dl_ values of 42.85, 53.11, and 31.59 mF cm^−2^, which were better than those of Os NPs (10.57, 5.18, and 5.22 mF cm^−2^) and Pt/C (24.23, 9.01, and 16.40 mF cm^−2^) in 1.0 M KOH, 0.5 M H_2_SO_4_ and 1.0 M PBS solutions, respectively, suggesting that the B-Os aerogel possessed more exposed electroactive sites than Os NPs and Pt/C^54^. Furthermore, the turnover frequency (TOF) was calculated to delve deeper into the inherent catalytic activity per site. Notably, the B-Os aerogel exhibited the highest TOF values of 1.17, 2.50, and 0.58 H^2^ s^−1^, which were about 4.88, 13.16, and 2.90 times as well as 2.05, 2.03, and 1.81 times higher than those of Os NPs (0.24, 0.19, and 0.20 H^2^ s^−1^) and Pt/C (0.57, 1.23, and 0.32 H^2^ s^−1^) at an overpotential of 100 mV in the alkaline solution, at an overpotential of 50 mV in the acidic solution and at an overpotential of 100 mV in the neutral solution, respectively (Fig. 3c). The TOF values of B-Os aerogels were much higher than those of Os NPs and Pt/C in the entire investigated potential range, suggesting a greater intrinsic capacity for electrocatalytic hydrogen production (Supplementary Fig. 17)^55^. As an indispensable evaluation criterion for promising electrocatalysts, the long-term stability of B-Os aerogels was further explored. As illustrated in Fig. 3d, e, the B-Os aerogel exhibited a negligible negative shift (2 mV) at a current density of 10 mA cm^−2^ in 1.0 M KOH solution, while the Os NPs and Pt/C catalysts demonstrated 39 and 24 mV negative shifts after 5000 cycles, respectively. Similarly, in 0.5 M H_2_SO_4_ and 1.0 M PBS solution, the polarization curves of B-Os aerogels before and after 5000 cycles also remained almost invariant relative to those of Os NPs and Pt/C (Fig. 3f and Supplementary Figs. 18 and 19). The excellent stability and durability were also confirmed by the chronoamperometry method. As exhibited in Fig. 3g, in pH-universal electrolytes, the B-Os aerogel showed negligible decay after 20 h compared with Os NPs and Pt/C. Besides, the TEM images before and after the stability tests indicated that there was no obvious change in the structure and morphology of B-Os aerogel, further suggesting their robustness (Supplementary Fig. 20). Subsequently, we also prepared the Os aerogels with different boron concentrations by varying the amount of the NaBH_4_ (Supplementary Table 5) and explored their HER performance in a 1.0 M KOH solution. As shown in Supplementary Fig. 21, at a current density of 10 mA cm^−2^, the overpotential of Os(B)−2 is 19 mV, which is better than Os(B)−1, Os(B)-3, and Os(B)-4. This result shows that the amount of B doping is critical to the HER performance of Os aerogels. In addition, we prepared other precious metal aerogels with similar methods and tested their performance in 1.0 M KOH for HER (Supplementary Fig. 22). As shown in Supplementary Fig. 23a, b, the B-Os aerogel required an overpotential of only 19 mV to generate the current density of 10 mA cm^−2^, which was much lower than those of Pt (33 mV), Rh (43 mV), Ru (45 mV) and other NMAs. Moreover, the B-Os aerogels showed the smallest Tafel slope and the highest C_dl_ among all the NMAs, indicating the optimal HER kinetics and the superior active surface area (Supplementary Figs. 24–26). Hence, these results proved that the B-Os aerogel outperforms the HER activity of the other NMAs (Supplementary Table 6). The superior electrocatalytic HER activity and stability can be attributed to the 3D porous ultra-fine network structure, abundant defect sites, surfactant-free clean surface and distinctive Os-B configuration, which provide highly accessible active sites, favorable mass/electron transfer channels and optimal electronic structure.

### Active sites identification

**Fig. 4:**
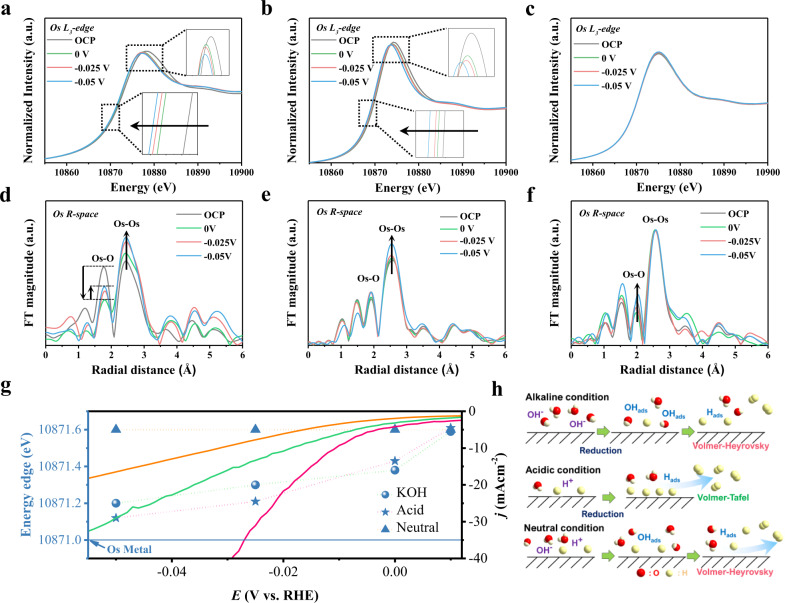
Operando investigation of HER behaviors in various electrolyte environments. **a** Operando XANES spectra of Os-B in 1.0 M KOH, **b** in 0.5 M H_2_SO_4_ and **c** in 1.0 M PBS. **d** Operando EXAFS spectra of Os-B in 1.0 M KOH, **e** in 0.5 M H_2_SO_4_ and **f** in 1.0 M PBS. **g** The polarization curve and the corresponding position of the Os energy edge obtained with a first-derivative method. **h** Schematic illustration of HER mechanism in various electrolyte environments.

The dynamic changes in the catalyst surface during the catalytic process have attracted much attention^36,39^. Here, we utilized operando XAS to probe the HER mechanism in various electrolyte environments. The operando XANES spectra were recorded under HER conditions to gain insight into the local variation of the electronic structure of the Os metal site. Figure 4a shows that the edge position of B-Os aerogels in the alkaline media was shifted to lower energy; the white-light intensity decreased, indicating the reduction of the Os electronic structure during the HER. Similar changes of B-Os aerogels in the acid media were also observed (Fig. 4b). Those phenomena suggested that the changes of the electronic structure of B-Os aerogels in both the alkaline and acid media facilitated electron transfer, thus promoting the kinetics during the HER. In contrast, the Os electronic state in the neutral media was not obviously altered with the applied potentials, indicating that the B-Os aerogels have self-adaptative behaviors during the catalytic process (Fig. 4c). Moreover, operando EXAFS measurements were employed to understand the local structures of Os sites reacting with water molecule species during the HER. Supplementary Fig. 27 shows the Os *L*-edge k^3^χ(k) oscillation functions of B-Os aerogels at various potentials. As shown in Fig. 4d, the increasing intensity of an Os-Os metal peak at 2.5 Å during the HER for B-Os aerogels indicated a significant reduction in the Os valence state. The Os-O peak (1.9 Å) at the OCP condition indicated the accumulation of OH species bonded on the catalyst surface. Notably, the decreasing intensity of Os-OH was observed on decreasing the applied potential, which was regarded as desorption of OH species on the Os sites, further triggering the processes of adsorption and desorption of intermediate H*. The operando EXAFS spectra of B-Os aerogels in the acid media in Fig. 4e shows the HER behavior different from those in the alkaline media. The intensity of the Os-Os metal peak gradually increased during the HER, indicating the formation of metallic-like Os. Meanwhile, the intensity of Os-O peak at 1.9 Å was not apparently changed, suggesting the proton may easily react on the catalyst surface in the acid media. This metallic-like Os with bare Os sites facilitated bonding with intermediate H* species, then formatting Os-H* through a Volmer step in the HER processes. In the neutral media, the operando EXAFS spectra of Os-Os peak in B-Os aerogels remained unchanged (Fig. 4f). However, the intensity of Os-O peak showed slightly increasing, regarding as the water molecules adsorbed on the Os sites so as to break H_2_O into intermediate H* reacted on the catalyst surface. As shown in Fig. 4g, the cathodic curve plotted with the Os energy edge indicated that no metallic Os^0^ (10871 eV) formed during the catalytic process, which suggests that the B content is beneficial to stabilize a metallic-like Os state. This slightly oxidized state under a cautious reduction potential could be vital to overcome the HER barrier^33,39^. Figure 4h exhibits the HER mechanisms of B-Os aerogels. In the alkaline condition, the B-Os aerogels underwent reduction to adapt the OH species to bond on the catalyst surface and further bond with H* through the Volmer–Heyrovsky step. The gradually decreased valence state in the acidic media also showed that B-Os aerogels interacted with H* in the Volmer–Tafel step. These results demonstrated the mechanisms for HER, implying that metallic-like Os was truly an active species for HER in both the alkaline and acidic media. In the neutral condition, the B-Os aerogels underwent a Volmer–Heyrovsky step and remained in a stable valence state, indicating that the B doping maintains the B-Os aerogel structure during the HER.

### Density functional theory (DFT) calculations

**Fig. 5:**
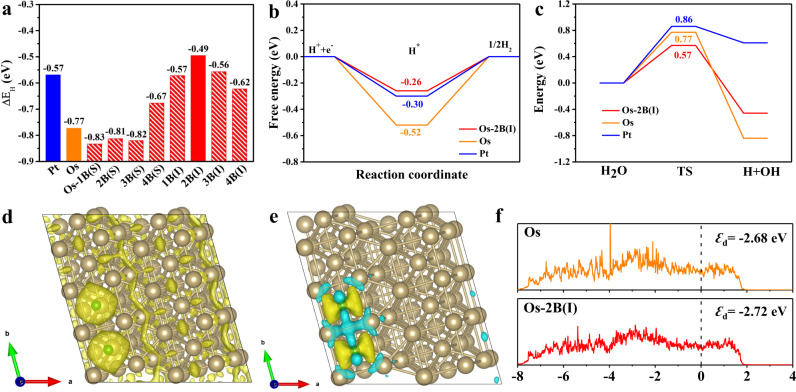
DFT calculations results. **a** The H adsorption energy for Pt, Os, and Os with different doping configurations. **b** Calculated Gibbs-energy diagram of HER at the equilibrium potential (U_RHE_ = 0 V) for Pt, Os, and Os-2B(I). **c** Calculated energy profile for water dissociation on the Pt (blue), Os (orange), and Os-2B(I) (red) surfaces. In **c**, the numbers represent the corresponding energy barriers in the units of eV. **d** Electron-localization function of Os-2B(I). **e** Charge-density difference plot of Os-2B(I). The yellow (blue) isosurface is drawn at the density of 0.01 (–0.01) e Å^–3^. The gold and green circles represent Os and B atoms, respectively. **f** The density of states (DOS) and *d*-band center values for Os and Os-2B(I). The zero-energy indicates the Fermi level.

To shed light on the origin of the enhanced HER performance of B-Os aerogels, we performed DFT calculations. Here, the B-Os aerogel was simulated using a slab geometry comprising five Os atomic layers. The topmost layer is doped with B, and both substitutional and interstitial B doping with various concentrations were considered (See Methods for more details). The lowest-energy B doping is an interstitial configuration, as shown in Supplementary Table 7 and Supplementary Fig. 28. Figure 5a displays the calculated H adsorption energy (∆E_H_) on the Pt, Os, and Os-B surfaces. The corresponding H adsorption structures are shown in Supplementary Fig. 29. For Pt, ∆E_H_ is –0.57 eV, consistent with the previously reported results^56^. Notably, all the considered interstitial doped configurations show the ∆E_H_ (–0.49~–0.62 eV) close to that of Pt. Os with two adjacent interstitial B dopants (Os-2B(I)) has the highest ∆E_H_ (–0.49 eV), indicating its weaker binding with H. Subsequently, we further calculated the hydrogen adsorption free energy (ΔG_H*_) of Os-2B(I) to evaluate its HER activity where the higher the ΔG_H*_, the weaker the hydrogen adsorption, and vice versa. Figure 5b indicates that the ΔG_H*_ of Os-2B(I) is closer (–0.26 eV) to the optimal criteria for HER (ΔG_H*_ = 0 eV) than the other considered substrates, supporting the experimental results that the B-Os aerogel shows better HER activity than Os NPs and Pt/C^57^. However, in alkaline and neutral electrolytes, the displayed HER performance is difficult to be fully reflected by ΔG_H*_ alone because the dissociation of water might impose additional obstacles to influence the overall reaction rate. Consequently, we further explored the kinetics of H_2_O dissociation processes from the Volmer step using the nudged elastic band method for Pt, Os, and Os-2B(I)^11^. Figure 5c and Supplementary Figs. 30–32 show the calculated energy profiles for the dissociation processes and the corresponding initial, transition, and final states, respectively. Notably, the Pt surface shows the highest water dissociation energy barrier (ΔE_B_) (0.86 eV), which is much higher than those for Os (0.77 eV) and Os-2B(I) (0.57 eV). The substantial reduction in water dissociation energy of the B-Os substrate is in favor of the formation of adsorbed H atoms to further generate H_2_. To deeply understand the origin of the enhanced HER performance, we investigate the regulative effect of interstitial B doping on the electronic configuration of B-Os aerogels. As shown in Fig. 5d, the electron-localization function for Os-2B(I) exhibits localized electrons centered at B atoms. The differential charge-density plot (Fig. 5e) and Bader charge analysis reveal that the B atoms gain electrons (0.07 *e*) from adjacent Os atoms, which is consistent with the aforementioned XPS and XANES analyses. This verified that the electronic structure of the metal center near the dopants was reformed, thus boosting the catalytic reaction kinetics. Figure 5f shows the *d*-band center (*ε*_d_) of Os and Os-2B(I) in the density of states. As expected, the introducing interstitial B atoms lower the *ε*_d_ of Os down from the Fermi level, manifesting a weaker H adsorption, which is in accordance with the *d*-band theory^58,59^. From the above results, it is believed that constructing B atoms can regulate the electronic configuration of Os, resulting in an improved ΔE_B_ and a more thermoneutral ΔG_H*_, and thus improve the HER activity.

## Discussion

In summary, we reported a facile strategy to synthesize interstitial B-Os aerogels with approximate 1.7 nm diameters and abundant defects, which serve as a highly efficient electrocatalyst for hydrogen evolution at all pH levels. The as-obtained B-Os aerogels show high electrocatalytic activities and superior stability for pH-universal HER, which are superior to commercial Pt/C and most reported noble-metal-based catalysts. Impressively, at the overpotential of 100 mV, the B-Os aerogels exhibit a 6.3-fold higher price activity (83.33 A dollar^−1^) than that of commercial Pt/C (13.32 A dollar^−1^) in the alkaline electrolyte. XANES and XPS results indicate that the introduced B atoms lead to the strong electron interaction between B and Os atoms and electron depletion on Os. DFT calculations also confirmed that the electronic configuration of Os is modulated by the interstitial doped B atoms, which can effectively lower ΔE_B_ and optimize good thermoneutral ΔG_H*_, thus significantly improving the HER performance. More importantly, the dynamic active-site construction and detailed catalytic mechanisms of B-Os catalysts during the electrocatalytic HER in the various electrolytes were identified by operando XAS, which shows that B can optimize the electron configurations of B-Os aerogels and stabilize osmium in an electron-deficient state during the catalytic reaction process. This work opens up further opportunities for the rational design of metal-based electrocatalysts with high performance via a heteroatom modification strategy for other energy conversion applications and beyond.

## Methods

### Chemicals

Osmium(III) chloride (OsCl_3_, 99.9%), sodium borohydride (NaBH_4_, 99.8%), osmium (Os, 99.9%), osmium tetroxide (OsO_4_, ≥98.0%), chloroplatinic acid hexahydrate (H_2_PtCl_6_·6H_2_O, ≥37.50% Pt basis), silver chloride (AgCl, 99%), ruthenium(III) chloride hydrate (RuCl_3_·xH_2_O, 99.98%) and hydrochloric acid (HCl, 37%) were purchased from Sigma-Aldrich. Gold chloride trihydrate (HAuCl_4_·3H_2_O, ≥99.9%), potassium hexachlororhodate(III) (K_3_RhCl_6_, Rh≥23.3%), iridium chloride (IrCl_3_, 99.8%) and palladium chloride (PdCl_2_, 99.999%) were purchased from Aladdin (Shanghai, China).

### Synthesis of B-Os aerogels

In a typical preparation of B-Os aerogels, OsCl_3_ (14.83 mg) was ultrasonically dispersed in H_2_O (1 mL), followed by mixing with 5 mL of freshly prepared NaBH_4_ aqueous solution with a certain concentration. The mixture was reacted for 30 min until the upper layer solution became transparent. The as-synthesized B-Os aerogels were washed six times using water and dried at 50 °C in a vacuum oven overnight. Different concentrations of NaBH_4_ (namely, 5 mM for Os(B)−1, 50 mM for Os(B)−2, 500 mM for Os(B)-3 and 5000 mM for Os(B)-4) were used. The other NMAs were prepared in a similar way only by replacing OsCl_3_ with the corresponding metal precursor.

### Characterization

The morphology and structure of the samples were measured by SEM (JSM-2010), TEM (JEOL JEM-2100), ACTEM (FEI Theims Z), and BET (MIC ASAP2460). XPS measurements were carried out using a Thermo Scientific K-Alpha spectrometer with a monochromatic Al Kα X-ray source. XRD patterns were collected on an X-ray diffractometer (Shimadzu, XRD-6000) with Cu-Kα radiation. The composition of the B-Os aerogel was measured by ICP-OES (PerkinElmer 8300).

### Electrochemical measurements

All electrochemical measurements were performed on an electrochemical workstation (CHI 660E) with a conventional three-electrode cell system. An L-type glassy carbon electrode (diameter: 3 mm, area: 0.071 cm^2^), saturated calomel electrode, and carbon rod were served as the working, reference, and counter electrodes, respectively. Before the electrochemical tests, the carbon-supported aerogels with the same metal loading content of 20% as electrocatalysts were synthesized. Typically, 2 mg as-prepared NMAs dissolved in 5 mL hexane and 8.0 mg Vulcan XC-72 carbon dissolved in 20 mL hexane were mixed under vigorous stirring for 12 h. The collected carbon-supported aerogels were redispersed in acetic acid, followed by heating at 70 °C for 3 h. The final catalysts were collected by centrifugation, and washed with ethanol three times. After drying, each catalyst (4 mg) was dispersed in the mixture including 0.235 mL isopropanol, 0.705 mL ethanol and 0.06 mL 5 wt% Nafion to form the well-mixed catalyst ink through 2 h of sonication. Five microliter of the suspension was dropped on an L-type glassy carbon electrode and dried at room temperature. Linear sweep voltammetry with IR compensation and CV were undertaken at a sweep rate of 2 mV s^−1^ in N_2_-saturated 1 M KOH, 1 M PBS, or 0.5 M H_2_SO_4_ electrolyte, respectively. To measure the electrochemical capacitance, CV was tested with varied scan rates, 40–160 mV s^−1^. Chronoamperometric characterization was implemented in the specified electrolyte at a current density of 10 mA cm^−2^ for 20 h. The reference electrode was calibrated in a highly pure hydrogen-saturated, and all the potentials in this work were converted to a reversible hydrogen electrode. The TOF was calculated with the following equation: TOF = *jA*/*n*F*N*, where *j* is the current density under a specified overpotential with iR correction, *A* is the geometric area of the L-type glassy carbon electrode (0.071 cm^−2^), *n* is the number of electrons transferred in the reaction (2 for HER), F is the Faraday constant (96,485.3 C mol^−1^), and *N* is the number of active sites (mol) calculated with the total mass loading.

### Operando hard X-ray absorption measurements

The Os *L*_3_-edge XAS was recorded in a transmission mode at TPS BL-44A beamline station in NSRRC. The catalyst powders (10 mg) of B-Os aerogels were dispersed in ethanol with Nafion solution (50 μL, 5 %, Sigma-Aldrich), then sonicated for 20 min. The catalyst ink was drop-cast onto a carbon cloth. Operando hard XAS was also recorded in a three-electrode setup with a self-assembly cell. Kapton tape was utilized to seal the operando cell window, which allowed X-rays to be transmitted through the window and the catalyst, and to reach the detector for operando XAS spectra recording.

### Computational details

First-principles DFT calculations were performed using the projector-augmented wave method^60^ and the Perdew–Burke–Ernzerhof exchange-correlation functional^61^ as implemented in the Vienna Ab-initio Simulation Package^62,63^. The plane-wave basis set with a cutoff energy of 550 eV was adopted in all the calculations. The 4 × 4 supercell with five atomic layers was constructed to simulate the Pt (111), Os (101), and Os-B (101) surfaces; during structural optimization, the atoms of the top two layers were allowed to relax until the force exerted on each atom was less than 0.02 eV/Å. The Brillouin zone was sampled using a 3 × 3 × 1 mesh for Pt (111), Os (101), and Os-B (101). For B doping, doping ratios of up to four dopants in the unit cell were considered. The free energy for H* adsorption on a catalyst surface has been widely used to evaluate both H* adsorption and H_2_ desorption^64–66^. The Gibbs free energy of the H adsorption was calculated as ∆G_H*_ = ∆E_H*_ + ∆E_ZPE_ − T∆S, where ∆E_H*_, ∆E_ZPE_, and ∆S represent the hydrogen adsorption energy, zero-point energy, and entropy difference defined by a formula ∆S = S(H*) – 1/2·S(H_2_), respectively. Here, S(H*) and S(H_2_) represent the entropy of the adsorbed H atom and H_2_ in the gas phase at standard conditions, respectively, and the former is approximately zero^67–70^.

## Supplementary information

Supplementary Information

## Data Availability

Full data supporting the findings of this study are available within the article and its Supplementary Information. The source data generated in this study are available in the figshare repository (10.6084/m9.figshare.18865934) (ref. ^71^). Additional data are available from the corresponding authors upon reasonable request. Source Data are provided with this paper.

## Source data

Source Data
