# Supplementary material for: Interstitial boron-triggered electron-deficient Os aerogels for enhanced pH-universal hydrogen evolution
Source: Nat Commun. 2022 Mar 3;13:1143. doi: 10.1038/s41467-022-28805-8 (PMC8894469; doi:10.1038/s41467-022-28805-8)
Supplement: Supplementary file 1 — Supplementary Information [file 41467_2022_28805_MOESM1_ESM.pdf]

Supplementary Information for

Interstitial Boron-Triggered Electron-Deficient Os Aerogels for Enhanced pH-Universal Hydrogen Evolution

Li et al.

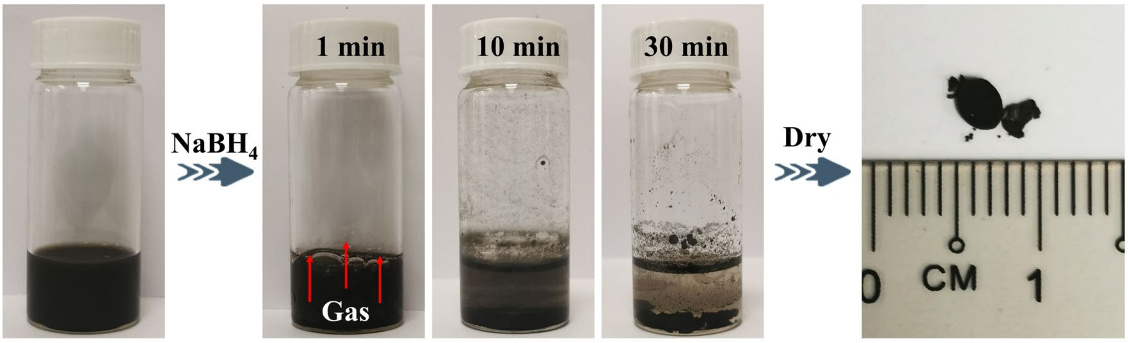

Supplementary Figure 1. Synthesis of catalysts. Images of the synthetic process of B-Os aerogels.

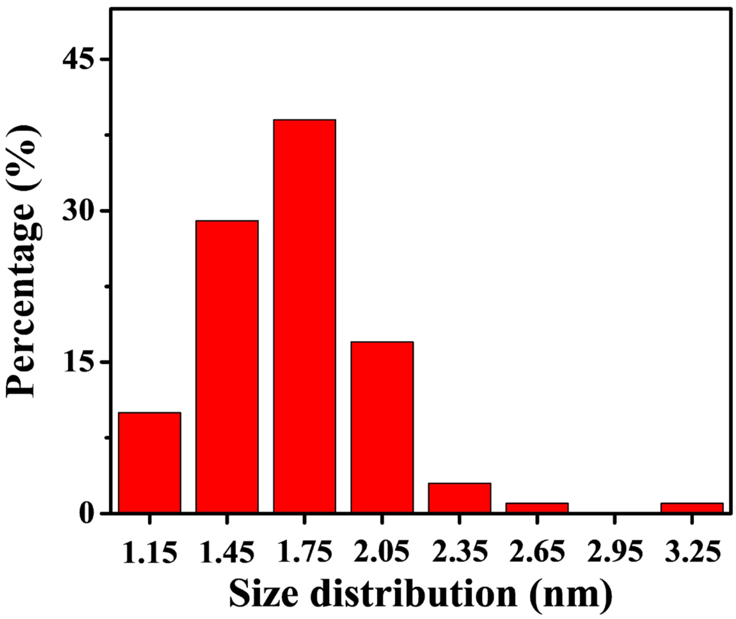

Supplementary Figure 2. Catalyst size. The size distribution of B-Os aerogels.

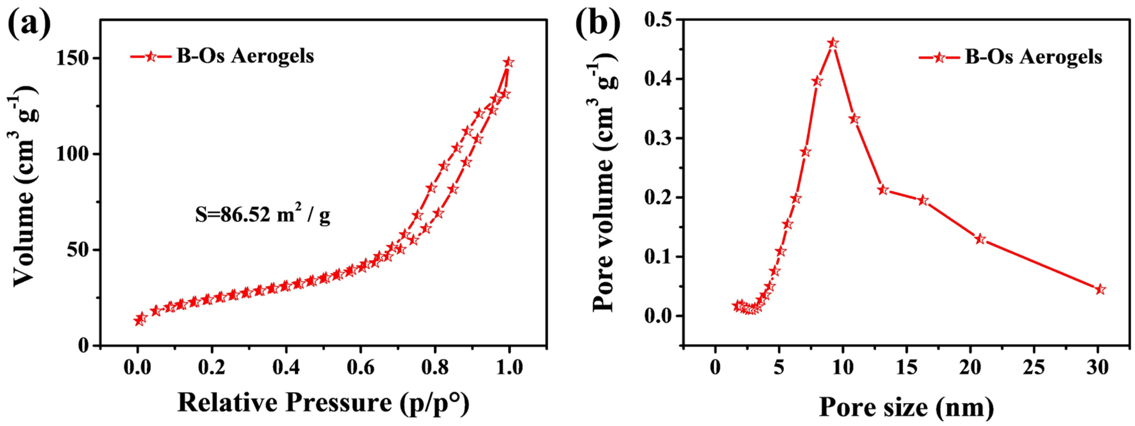

Supplementary Figure 3. Nitrogen adsorption tests of B-Os aerogels. (a) Adsorption/desorption isotherms and **(b)** Pore size distributions derived from the density functional theory as implemented in the instrument's software.

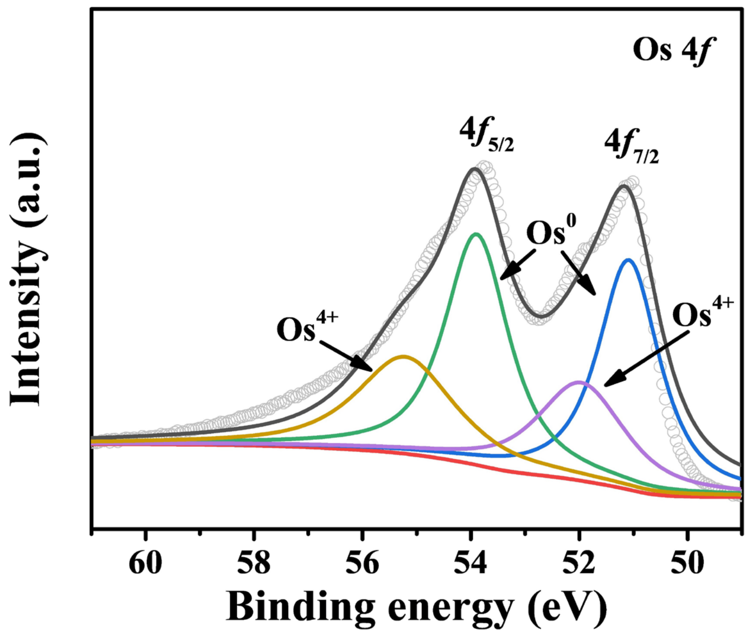

Supplementary Figure 4. XPS spectra. Os 4*f* XPS spectra of B-Os aerogels.

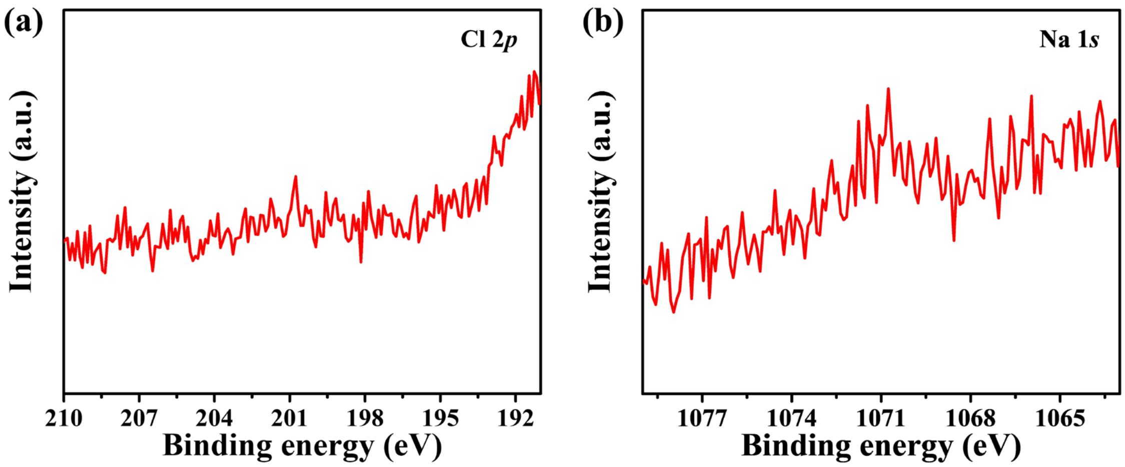

Supplementary Figure 5. XPS spectra. (a) Cl 2*p* XPS spectrum. **(b)** Na 1*s* XPS spectrum.

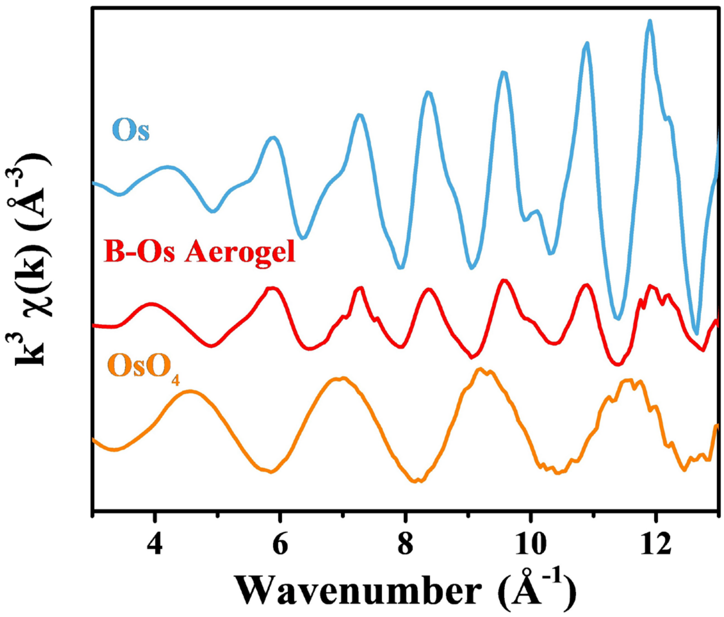

Supplementary Figure 6. XAS analysis. Os *K*-space oscillation, weighted by k^3.

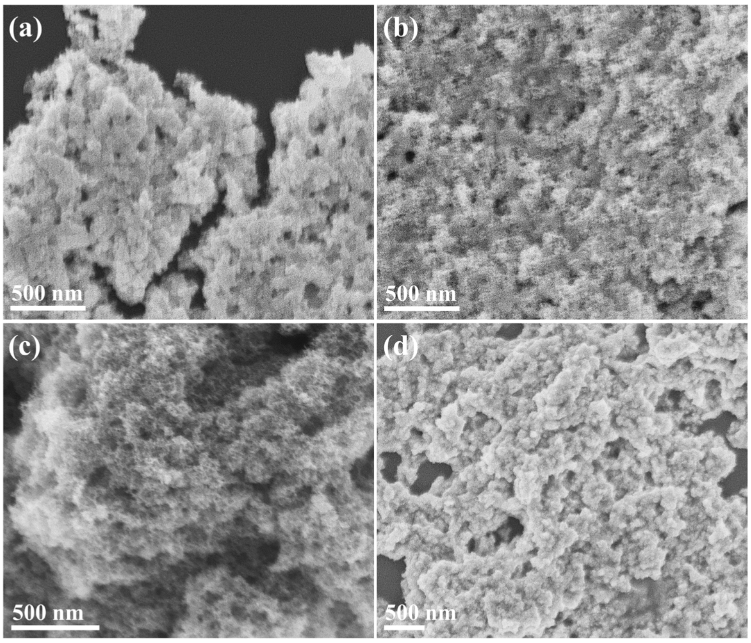

Supplementary Figure 7. SEM images of the samples prepared with different concentrations of metallic precursor under the identical synthesis conditions. (a) $NaBH_4$ (5 mL, 50 mM), $OsCl_3$ (1 mL, 1 mM); **(b)** $NaBH_4$ (5 mL, 50 mM), $OsCl_3$ (1 mL, 10 mM); **(c)** $NaBH_4$ (5 mL, 50 mM), $OsCl_3$ (1 mL, 50 mM); and **(d)** $NaBH_4$ (5 mL, 50 mM), $OsCl_3$ (1 mL, 100 mM).

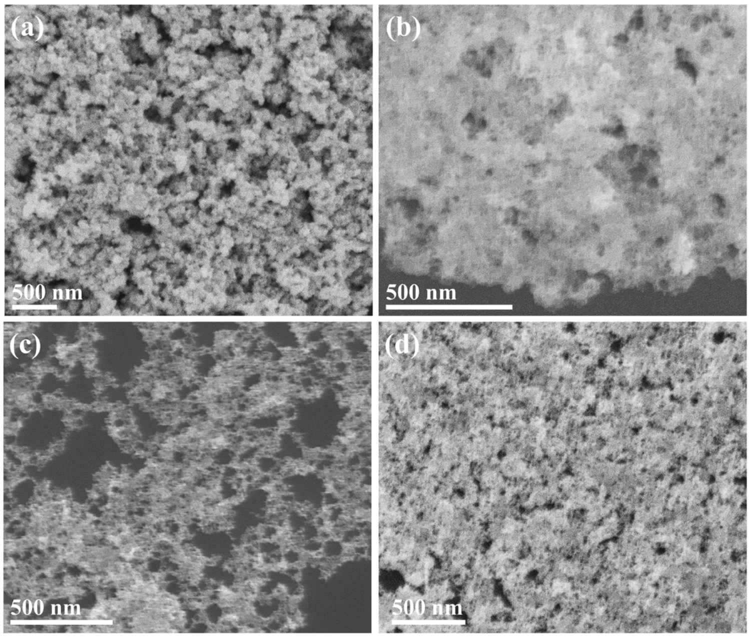

Supplementary Figure 8. SEM images of the samples prepared with different ratios of metallic precursor under the identical synthesis conditions. (a) $NaBH_4$ (2 mL, 50 mM), $OsCl_3$ (1 mL, 50 mM); **(b)** $NaBH_4$ (1 mL, 50 mM), $OsCl_3$ (1 mL, 50 mM); **(c)** $NaBH_4$ (5 mL, 50 mM), $OsCl_3$ (1 mL, 50 mM); and **(d)** $NaBH_4$ (10 mL, 50 mM), $OsCl_3$ (1 mL, 50 mM).

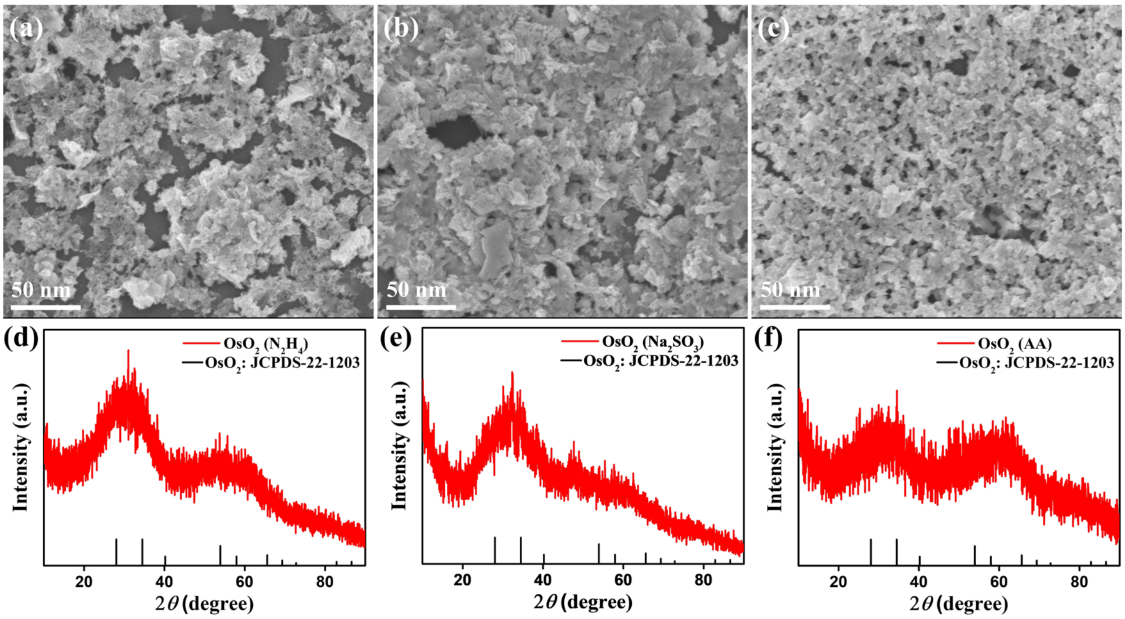

Supplementary Figure 9. SEM images and XRD patterns of samples fabricated by various reducing agents under the same synthesis conditions. (a, c) Hydrazine hydrate. **(b, d)** Sodium sulfite. **(c, f)** Ascorbic acid (AA).

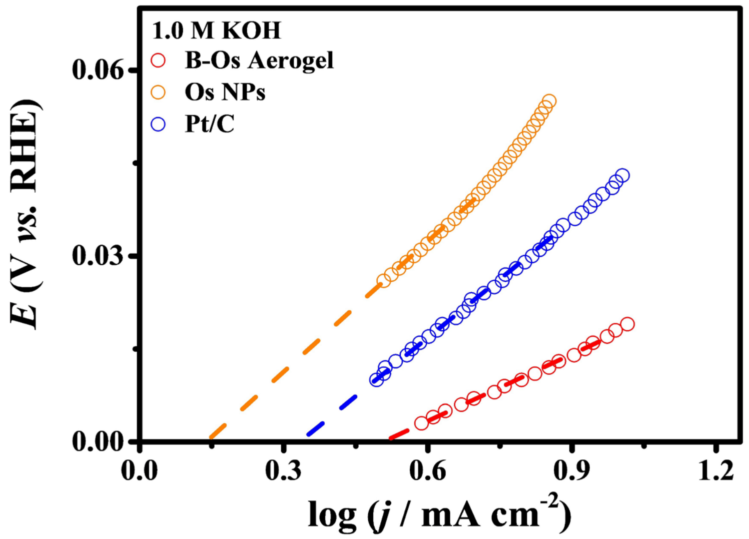

Supplementary Figure 10. Exchange current density analysis. Exchange current densities of B-Os aerogels, Os NPs and Pt/C calculated from Tafel plots by extrapolation method in 1.0 M KOH for HER.

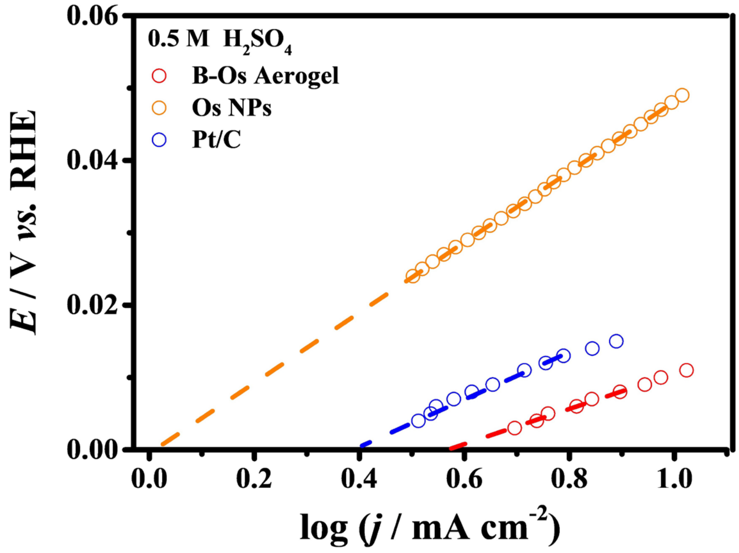

Supplementary Figure 11. Exchange current density analysis. Exchange current densities of B-Os aerogels, Os NPs and Pt/C calculated from Tafel plots by extrapolation method in 0.5 M H_2SO_4 for HER.

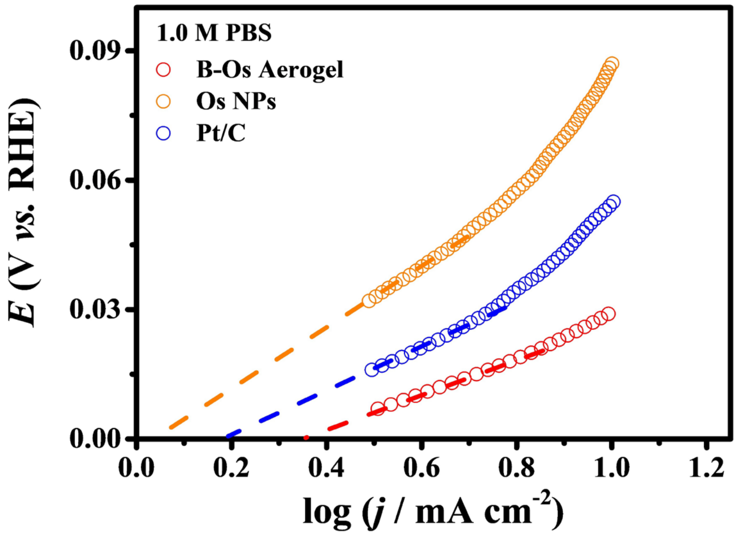

Supplementary Figure 12. Exchange current density analysis. Exchange current densities of B-Os aerogels, Os NPs and Pt/C calculated from Tafel plots by extrapolation method in 1.0 M PBS for HER.

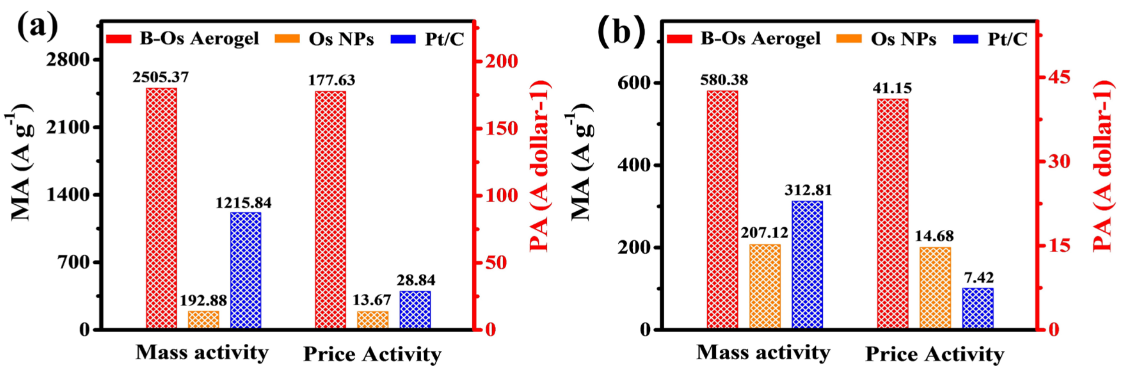

Supplementary Figure 13. Mass activity (MA) and price activity (PA) of B-Os aerogels, Os NPs and Pt/C. (a) At an overpotential of 50 mV in 0.5 M H_2SO_4 solution and **(b)** at an overpotential of 100 mV in 1.0 M PBS solution.

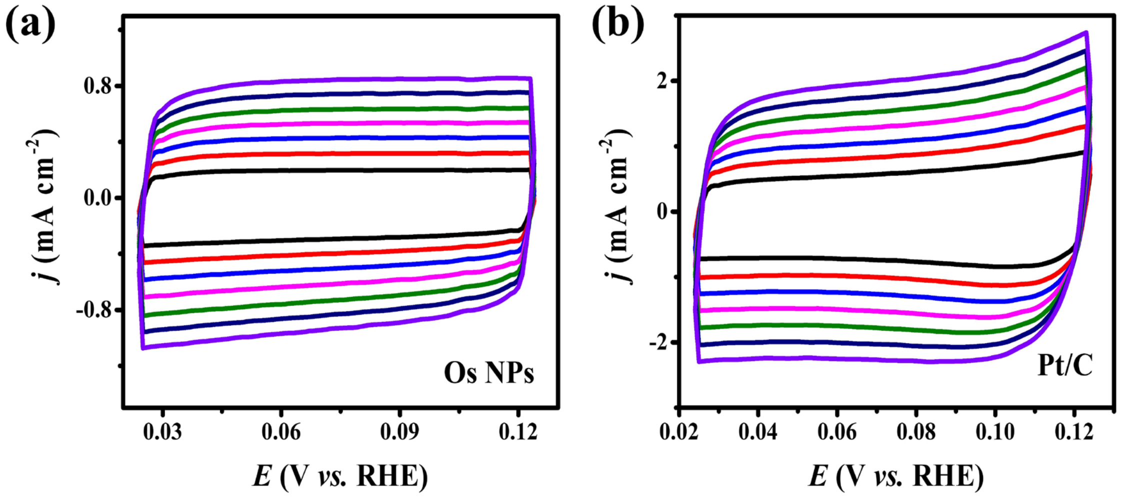

Supplementary Figure 14. CV plots in 1.0 M KOH solution. CV plots of **(a)** Os NPs and **(b)** Pt/C with varying scan rates from 40 to 160 mV s^{-1} in 1.0 M KOH solution.

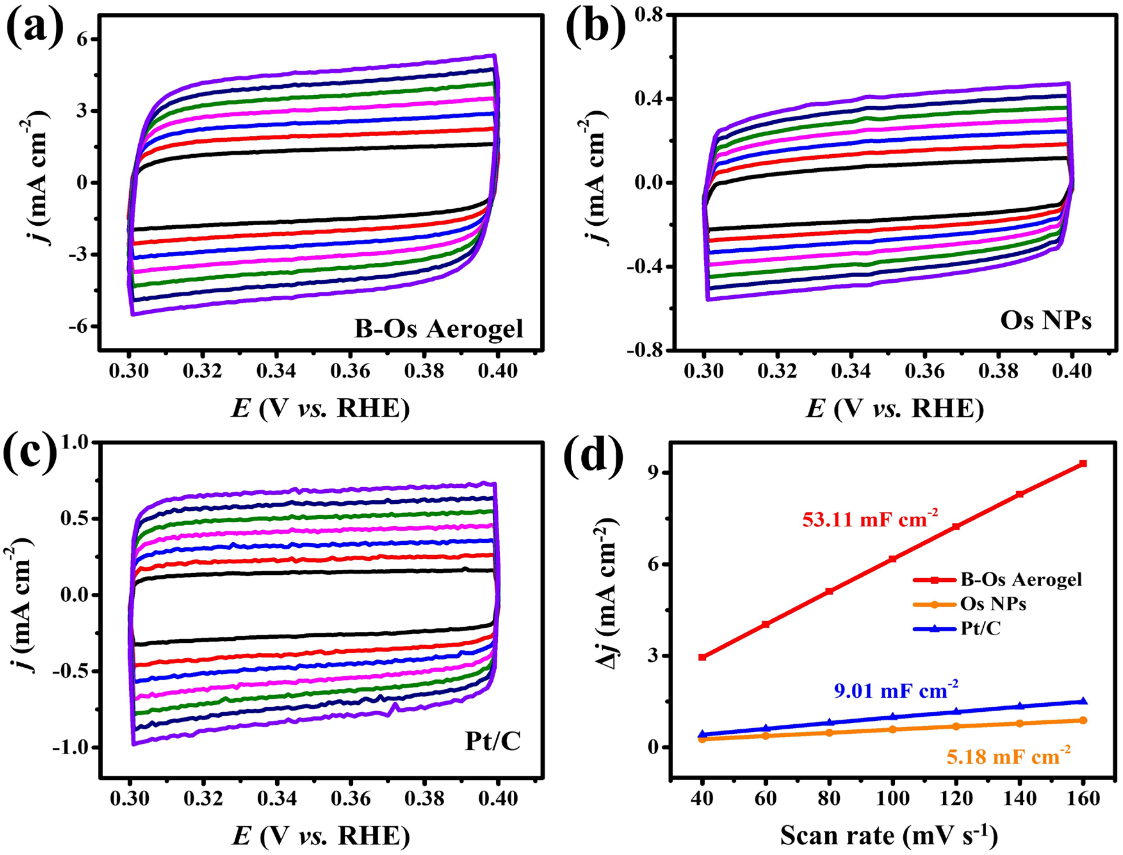

Supplementary Figure 15. CV plots and C_{dl} in 0.5 M H_2SO_4 solution. CV plots of **(a)** B-Os aerogels, **(b)** Os NPs and **(c)** Pt/C at varying scan rates from 40 to 160 mV s^{-1}. **(d)** C_{dl} of B-Os aerogels, Os NPs and Pt/C.

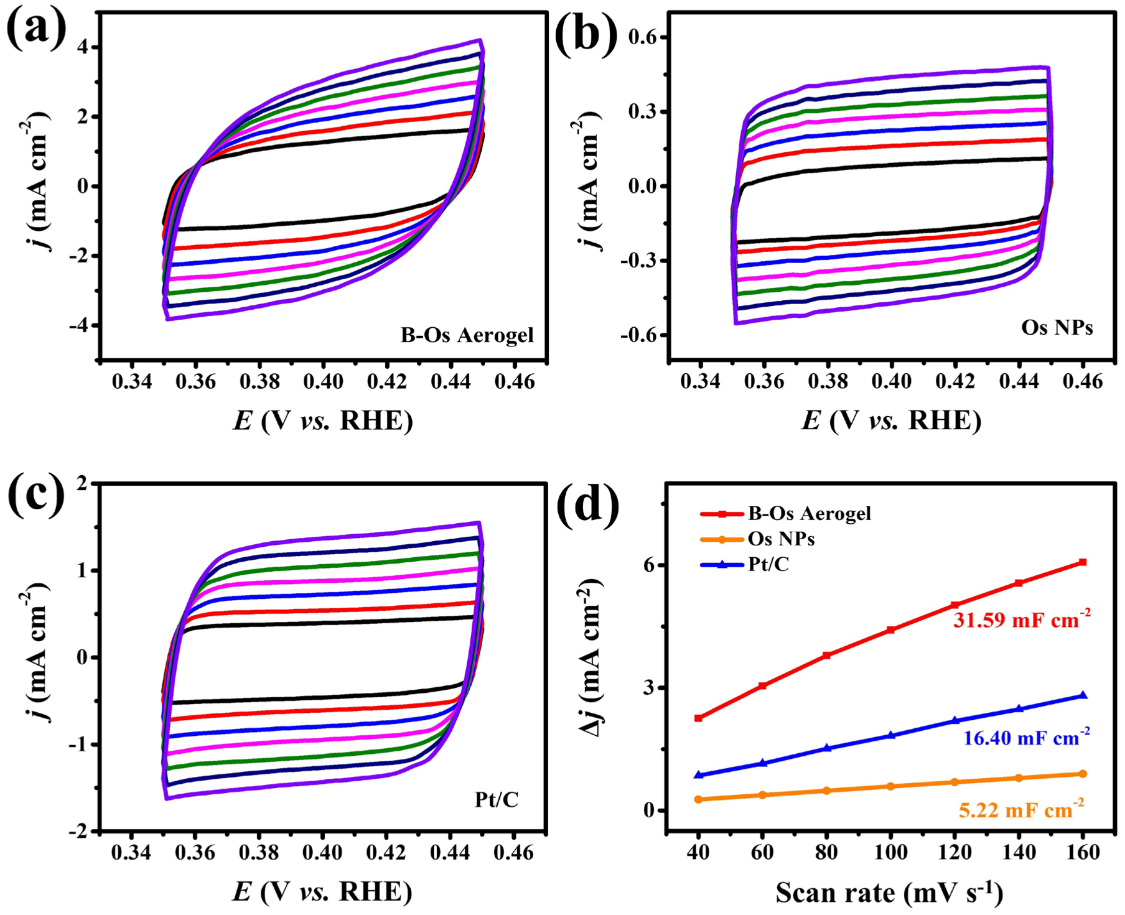

Supplementary Figure 16. CV plots and C_{dl} in 1.0 M PBS solution. CV plots of **(a)** B-Os aerogels, **(b)** Os NPs and **(c)** Pt/C at varying scan rates from 40 to 160 mV s^{-1}. **(d)** C_{dl} of B-Os aerogels, Os NPs and Pt/C.

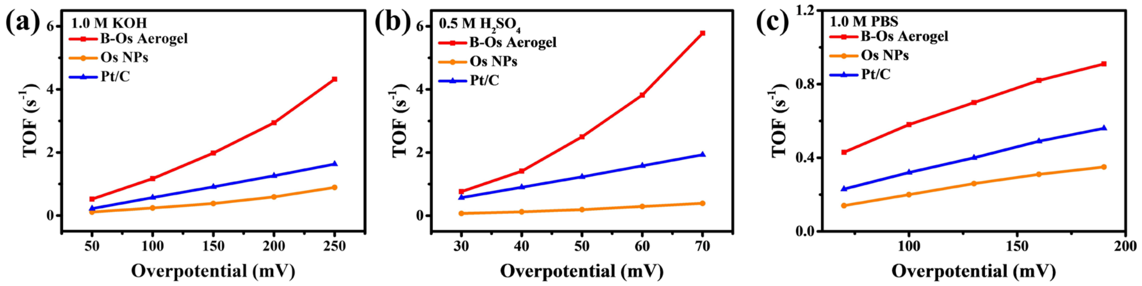

Supplementary Figure 17. TOF values of B-Os aerogels, Os NPs and Pt/C. **(a)** at an overpotential of 100 mV in 1.0 M KOH solution, **(b)** at an overpotential of 50 mV in 0.5 M H_2SO_4 solution and **(c)** at an overpotential of 100 mV in 1.0 M PBS solution.

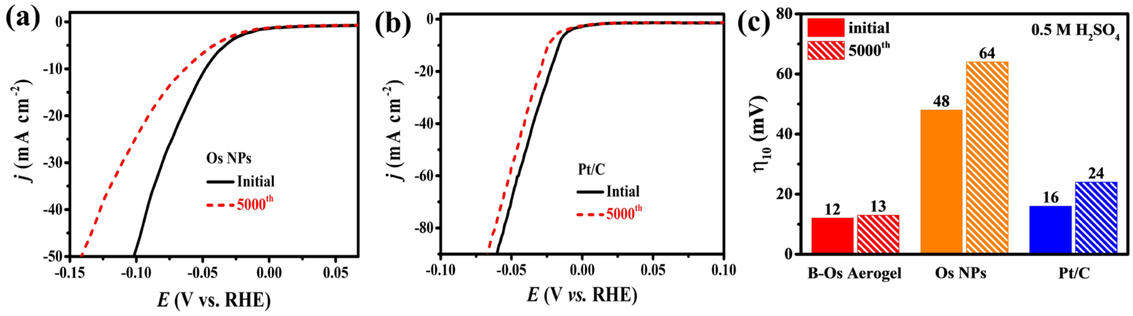

Supplementary Figure 18. Stability test in 0.5 M H_2SO_4 solution. iR-corrected polarization curves of **(a)** Os NPs and **(b)** Pt/C at 10 mA cm^{-2} in 0.5 M H_2SO_4 solution before and after 5000 CV potential cycles. **(c)** The corresponding overpotential changes of B-Os aerogels, Os NPs and Pt/C.

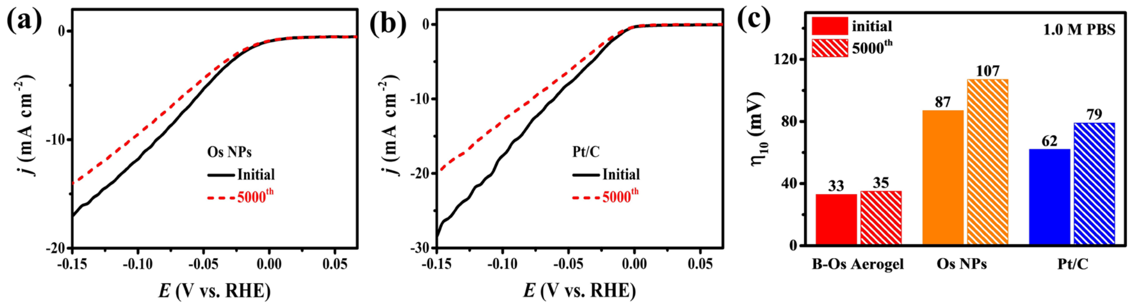

Supplementary Figure 19. Stability test in 1.0 M PBS solution. iR-corrected polarization curves of **(a)** Os NPs and **(b)** Pt/C at 10 mA cm^{-2} in 1.0 M PBS solution before and after 5000 CV potential cycles. **(c)** The corresponding overpotential changes of B-Os aerogels, Os NPs and Pt/C.

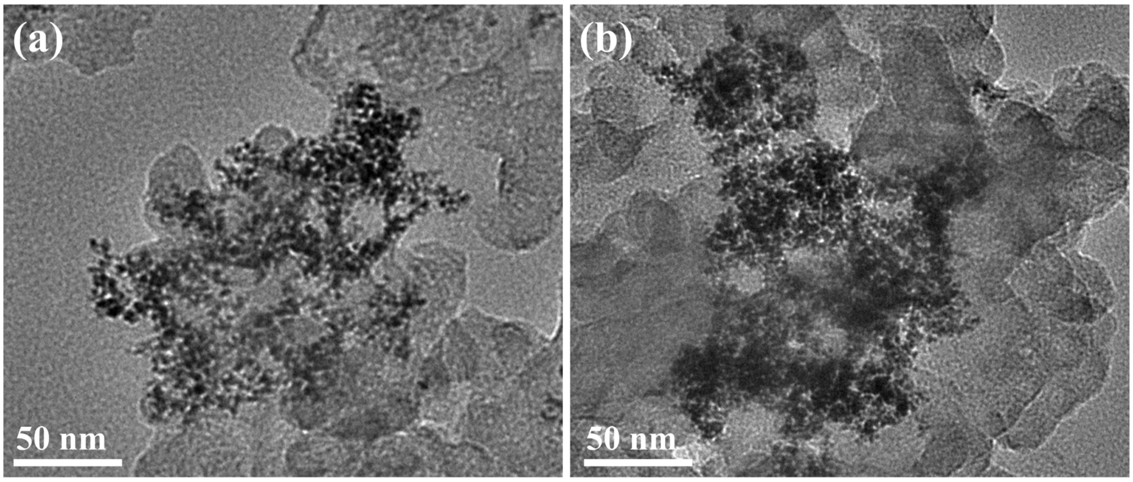

Supplementary Figure 20. TEM images of B-Os aerogels after stability test. (a) before HER, **(b)** after 20 h electrocatalytic stability test at a current density of 10 mA cm^{-2} in 1 M KOH.

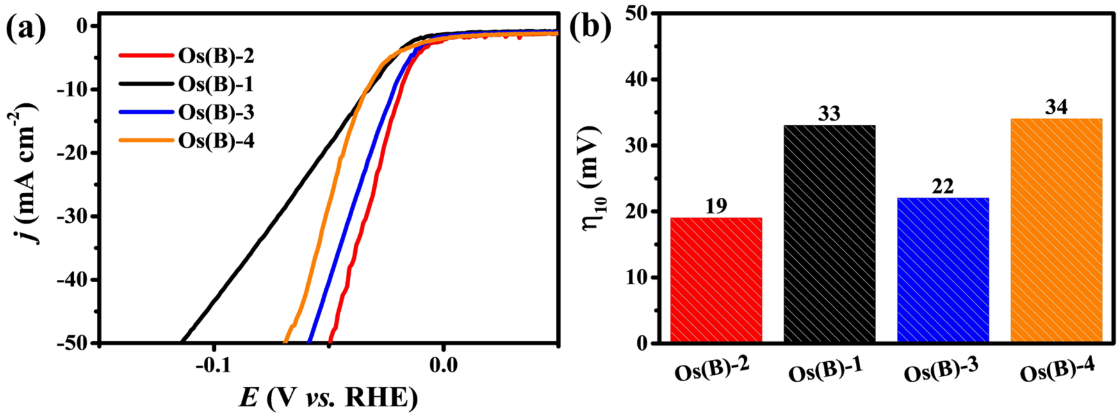

Supplementary Figure 21. Electrochemical HER performance measurements of B-Os aerogels with different boron concentration. **(a)** iR-corrected polarization curves. **(b)** The overpotential at 10 mA cm^{-2} in 1.0 M KOH solution.

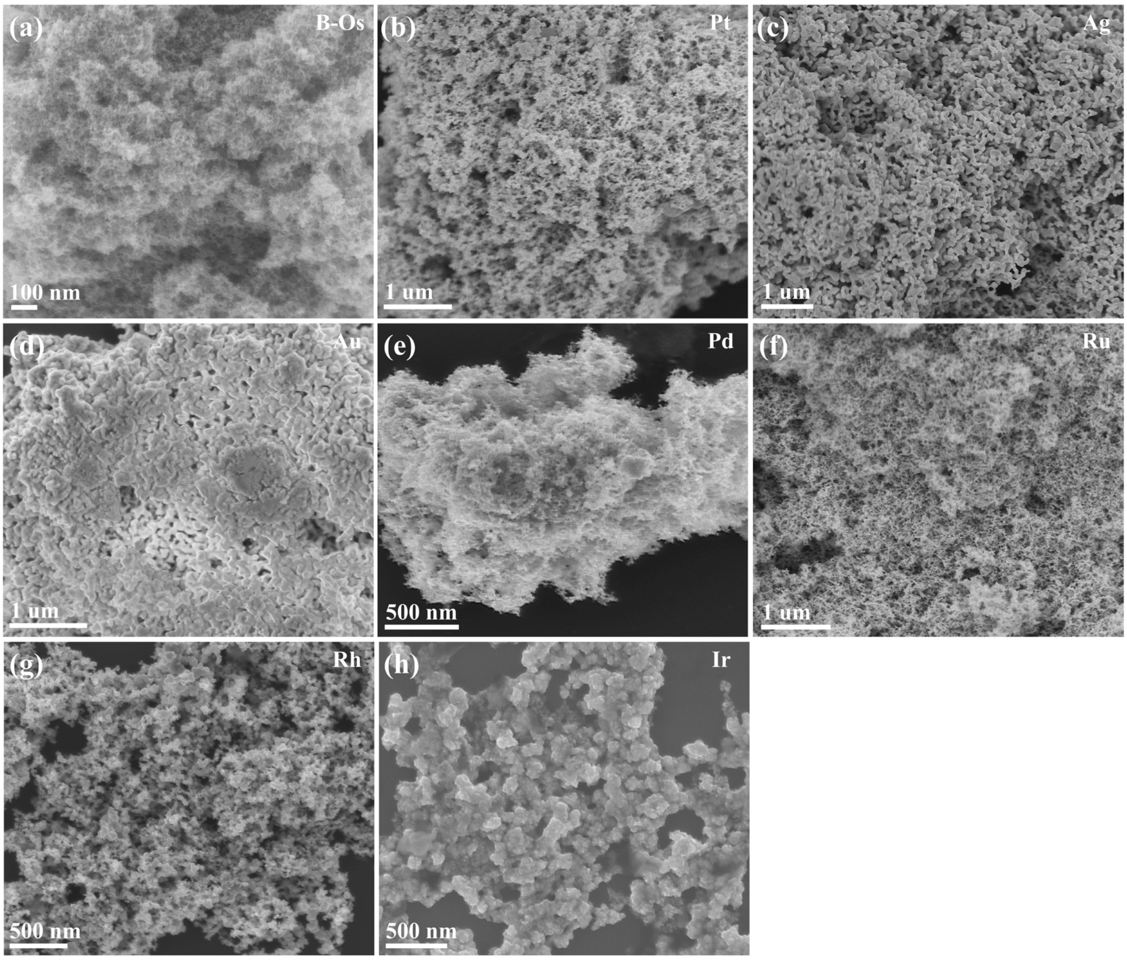

Supplementary Figure 22. SEM images of different noble metal aerogels. (a) B-Os, **(b)** Pt, **(c)** Ag, **(d)** Au, **(e)** Pd, **(f)** Ru, **(g)** Rh and **(h)** Ir.

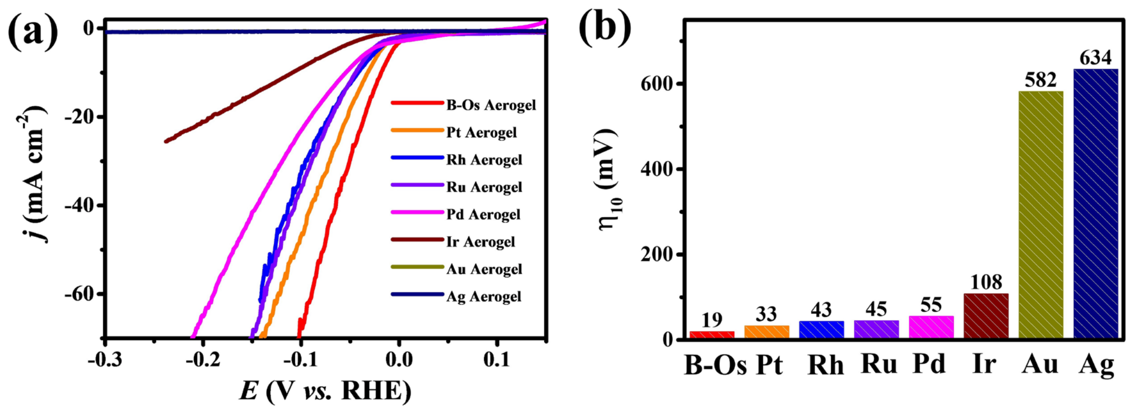

Supplementary Figure 23. HER performance of different noble metal aerogels. (a) iR-corrected polarization curves and **(b)** corresponding overpotential at 10 mA cm^{-2} in 1.0 M KOH solution.

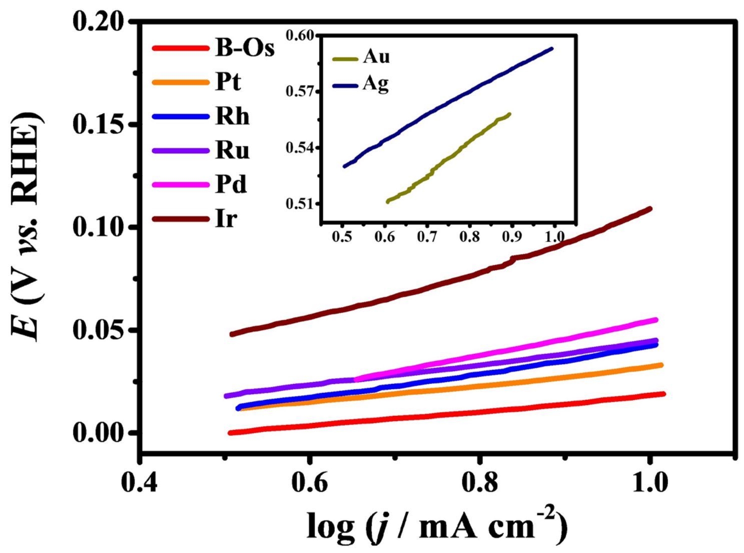

Supplementary Figure 24. Tafel slope analysis. Tafel plots of different noble metal aerogels in 1.0 M KOH solution.

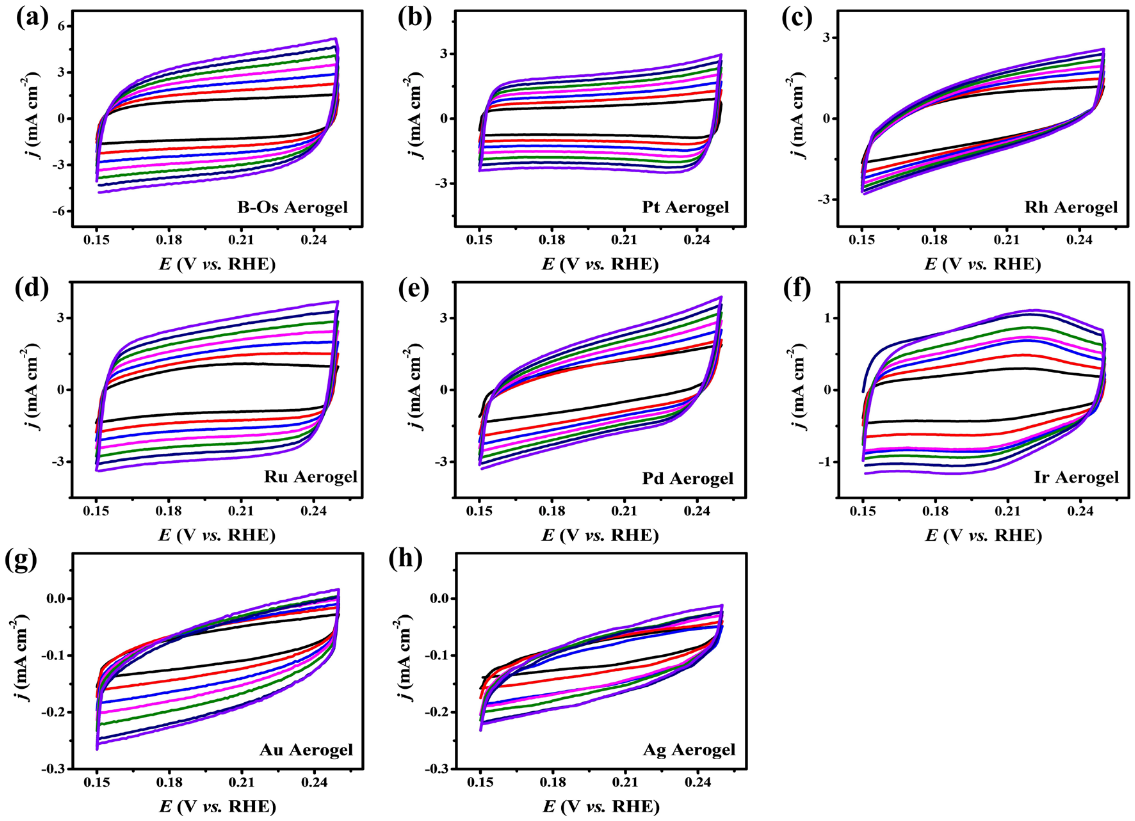

Supplementary Figure 25. CV plots of different noble metal aerogels. CV plots of **(a)** B-Os, **(b)** Pt, **(c)** Rh, **(d)** Ru, **(e)** Pd, **(f)** Ir, **(g)** Au and **(h)** Ag at varying scan rates from 40 to 160 mV s^{-1} in 1.0 M KOH solution.

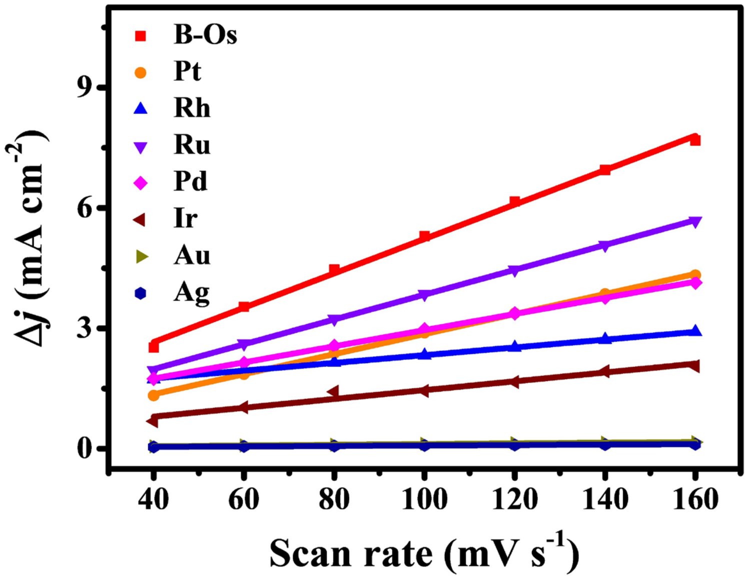

Supplementary Figure 26. C_{dl} analysis. C_{dl} of B-Os, Pt, Rh, Ru, Pd, Ir, Au and Ag aerogels in 1.0 M KOH solution.

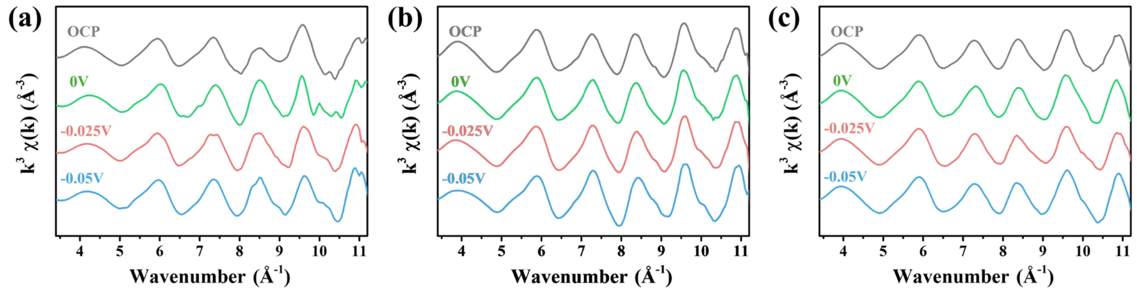

Supplementary Figure 27. XAS analysis. Os *K*-space oscillation, weighted by k3 **(a)** in alkaline, **(b)** acidic, and **(c)** neutral electrolytes, respectively.

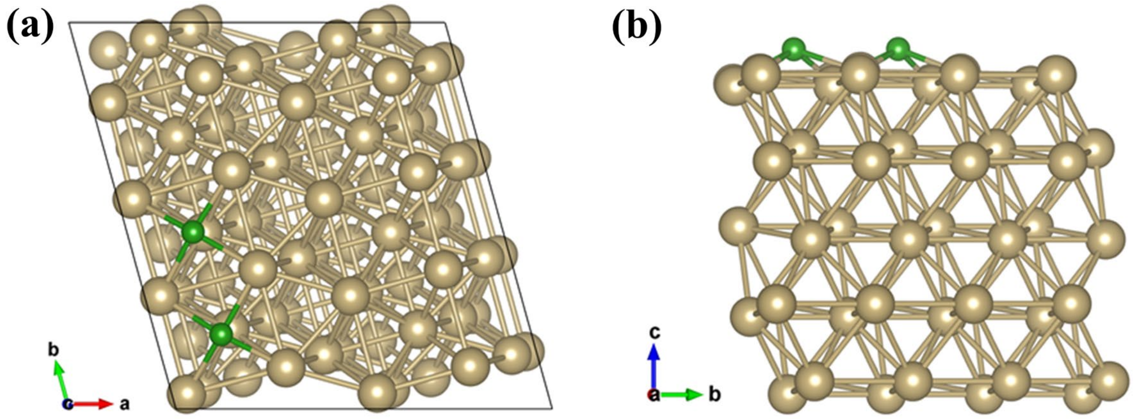

Supplementary Figure 28. DFT calculation model. (a) Top and **(b)** side view of the lowest-energy B interstitial doping configuration.

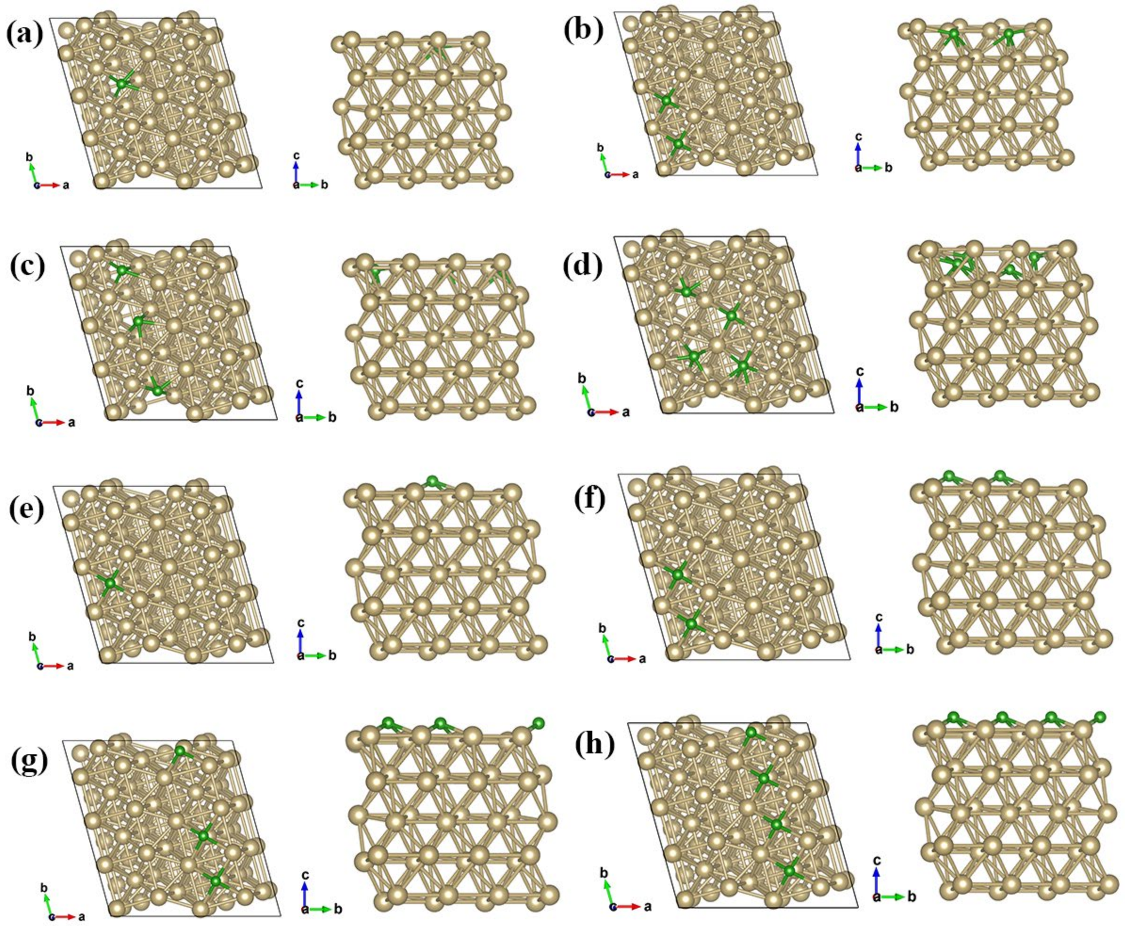

Supplementary Figure 29. DFT calculation model. Top and side view of the lowest-energy doping configurations for **(a)** Os-1B(S); **(b)** Os-2B(S); **(c)** Os-3B(S); **(d)** Os-4B(S); **(e)** Os-1B(I); **(f)** Os-2B(I); **(g)** Os-3B(I) and **(h)** Os-4B(I). The gold and green circles represent Os and B atoms, respectively.

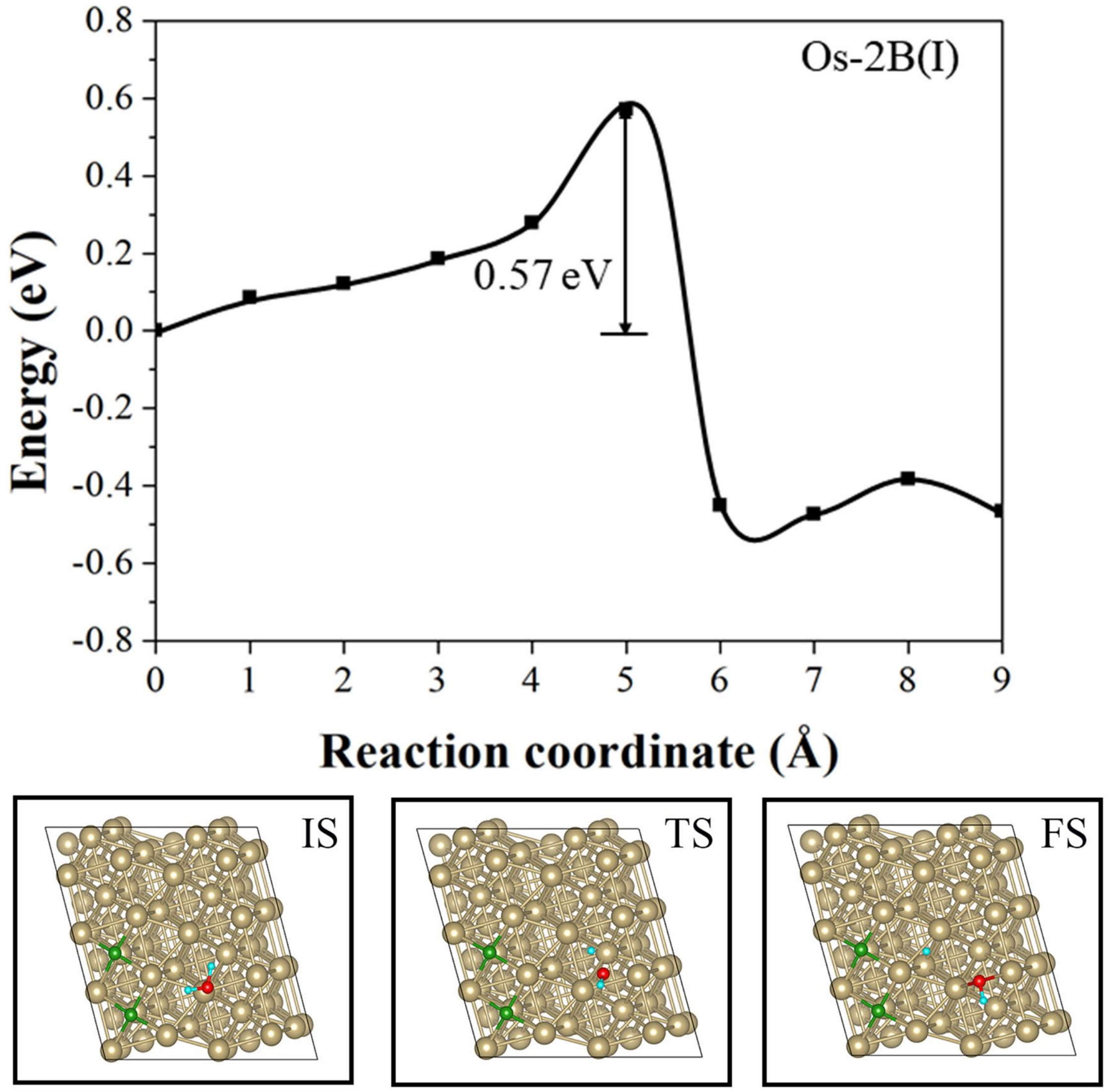

Supplementary Figure 30. The NEB calculated energy profile of Os-2B(I) for water dissociation on surfaces. The atomic geometries of the initial, transition and final states are also given. The gold, green, red and blue circles represent Os, B, O and H atoms, respectively.

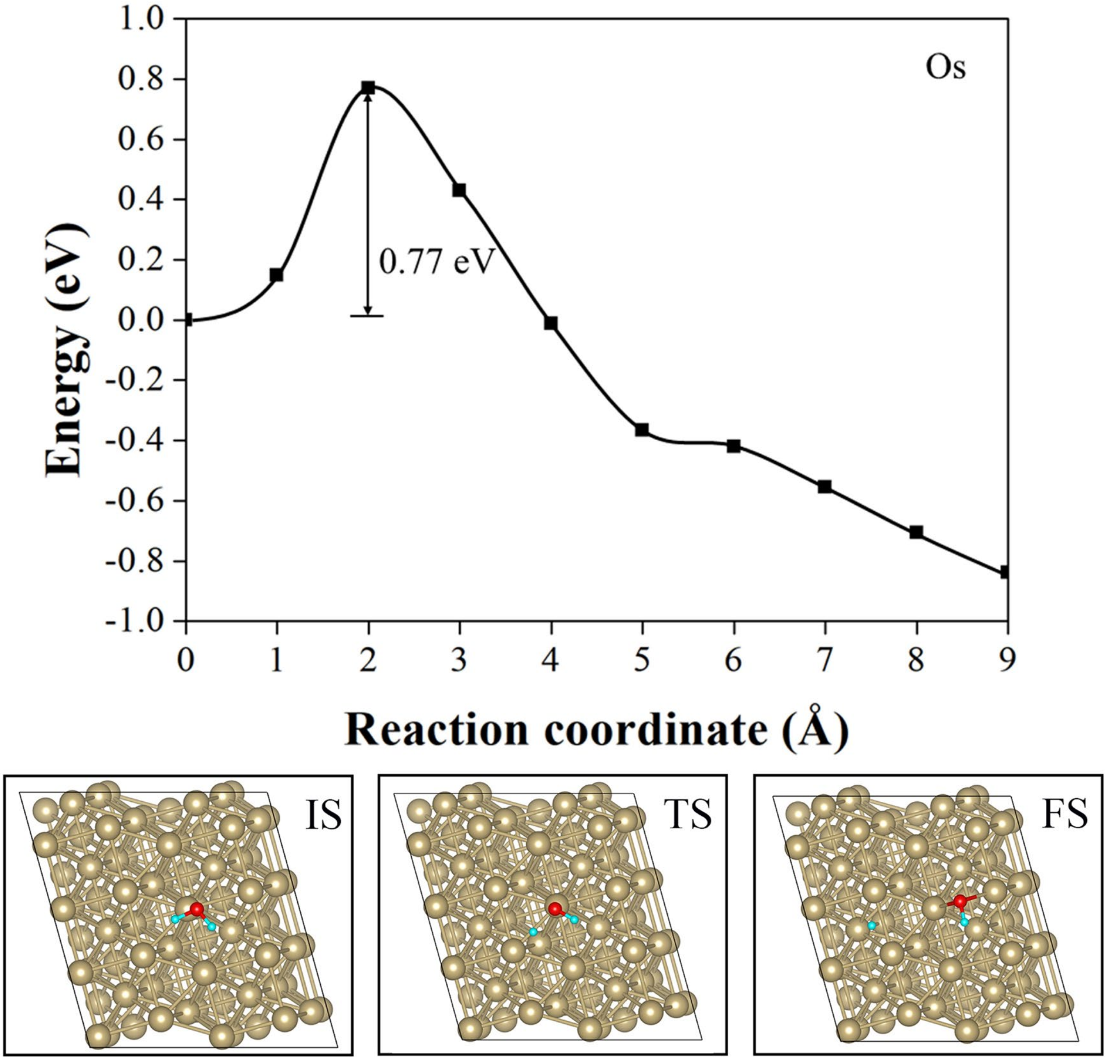

Supplementary Figure 31. The NEB calculated energy profile of Os for water dissociation on surfaces. The atomic geometries of the initial, transition and final states are also given. The gold, red and blue circles represent Os, O and H atoms, respectively.

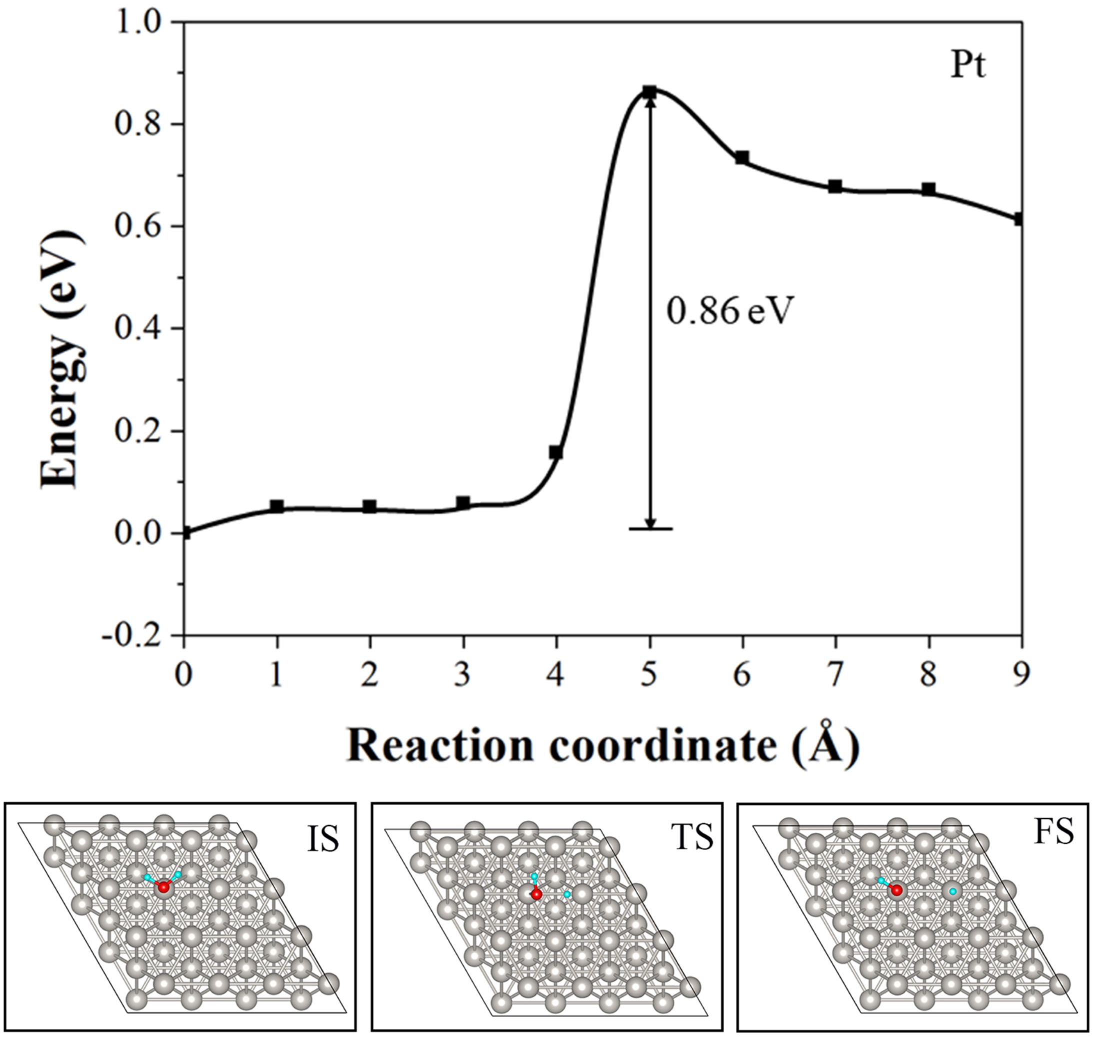

Supplementary Figure 32. The NEB calculated energy profile of Pt for water dissociation on surfaces. The atomic geometries of the initial, transition and final states are also given. The grey, red and blue circles represent Pt, O and H atoms, respectively.

Supplementary Table 1. Price of different noble metals.[a]

Metal	Symbol	Unit of Measure	U.S.
Platinum	Pt	troy ounce	$1094.00
Palladium	Pd	troy ounce	$2820.00
Rhodium	Rh	troy ounce	$17750.00
Iridium	Ir	troy ounce	$5500.00
Ruthenium	Ru	troy ounce	$750.00
Osmium	Os	troy ounce	$400.00
Gold	Au	troy ounce	$1804.00
Silver	Ag	troy ounce	$25.95

[a]The prices for various noble metals are from the BASF corporation website on July 12, 2021. (https://apps.catalysts.basf.com/apps/eibprices/mp/)

Supplementary Table 2. Summary of recently reported catalysts for HER in alkaline electrolyte.

Catalyst	Electrolyte	Overpotential @10 mA cm^{-2}	Tafel slope /mV.dec^{-1}	Ref.
**B-Os Aerogel**	**1.0 M KOH**	**19**	**35.8**	**This work**
**Pt/C**	**1.0 M KOH**	**43**	**51.5**	**This work**
$Ru@C_2N$	1.0 M KOH	17	38	Nat. Nanotechnol. 12, 441-446 (2017)
Ru@MWCNT	1.0 M KOH	17	27	Nat. Commun. 11, 1-10 (2020)
Ir-NSG	1.0 M KOH	18.5	28.3	Nat. Commun. 11, 1-10 (2020)
Ru@GnP	1.0 M KOH	22	28	Adv. Mater. 30, 1803676 (2018)
RuCoP	1.0 M KOH	23	37	Energy Environ. Sci. 11, 1819-1827 (2018)
WO_x-PtNi@Pt DNWs/C	0.1 M KOH	24	N/A	Adv. Energy Mater. 11, 2003192 (2020)
RP-CPM	1.0 M KOH	24	47.3	Sci. Adv. 6, eabb4197 (2020)
RuCo/C	1 M KOH	28	31	Nat. Commun. 8, 1-12 (2017)
$IrP_2@NC$	1.0 M KOH	28	50	Energy Environ. Sci. 12, 952-957 (2019)
Rh_2P	1.0 M KOH	30	50	Adv. Energy Mater. 8, 1703489 (2018)
$Ni@Ni_2P$-Ru	1.0 M KOH	31	41	J. Am. Chem. Soc. 140, 2731-2734 (2018)
Ru@CN	1.0 M KOH	32	53	Energy Environ. Sci. 11, 800-806 (2018)
$NiRu_{0.13}$-BDC	1.0 M KOH	34	32	Nat. Commun. 12, 1-8 (2021)
h-$RuSe_2$	1.0 M KOH	34	95	Angew. Chem. Int. Ed. 133, 7089-7093 (2021)
PdIr bimetallene/C	0.1 M KOH	34	58.3	Natl. Sci. Rev. 0, nwab019 (2021)

a-$RuTe_2$ PNRs	1.0 M KOH	36	36	Nat. Commun. 10, 1-11 (2019)
RhPd-H NPs	1.0 M KOH	36.6	35.3	ACS Nano 13, 12987-12995 (2019)
Pt-Ni-O	1.0 M KOH	39.8	78.8	J. Am. Chem. Soc. 140, 9046-9050 (2018)
RhPd-H	1.0 M KOH	40	35.7	J. Am. Chem. Soc. 142, 3645-3651 (2020)
Pt_3Ni_2-NWs-S/C	1.0 M KOH	42	N/A	Nat. Commun. 8, 1-9 (2017)
Rh NSs	1.0 M KOH	43	107.2	Chem. Mater. 29, 5009-5015 (2017)
Pt_1/N-C	1.0 M KOH	46	36.8	Nat. Commun. 11, 1-8 (2020)
Pt_5/HMCS	1.0 M KOH	46.2	48.1	Adv. Mater. 32, 1901349 (2020)
Ni-Pt-islands	0.1 M KOH	49	69	J. Am. Chem. Soc. 141, 16202-16207 (2019)
Au-Ru	1.0 M KOH	50	30.8	Nat. Chem. 10, 456-461 (2018)
Ni_3N/Pt	1.0 M KOH	50	36.5	Adv. Energy Mater. 7, 1601390 (2017)
RuP_2@NPC	1.0 M KOH	52	69	Angew. Chem. Int. Ed. 56, 11559-11564 (2017)
Ni_5P_4-Ru	1.0 M KOH	54	52	Adv. Mater. 32, 1906972 (2020)
$Ru_{0.33}Se$ @ TNA	1.0 M KOH	57	50	Small 14, 1802132 (2018)
2.20wt% Ru SAs-Ni_2P	1.0 M KOH	57	75	Nano Energy 80, 105467 (2021)
Sr_2RuO_4	1.0 M KOH	61	51	Nat. Commun. 10, 1-9 (2019)
hcp Pt-Ni NMPs	0.1 M KOH	65	78	Nat. Commun. 8, 1-7 (2017)
SANi-Pt NWs	1.0 M KOH	70	60.3	Nat. Catal. 2, 495-503 (2019)
Pt NWs/SL-$Ni(OH)_2$	1.0 M KOH	70	72	Nat. Commun. 6, 1-8 (2015)

OsP_2@NPC	1.0 M KOH	70	67	J. Catal. 370, 404-411 (2019)
Li-$IrSe_2$	1 M KOH	72	N/A	Angew. Chem. Int. Ed. 131, 14906-14911 (2019)
Ru/C_3N_4/C	1.0 M KOH	79	N/A	J. Am. Chem. Soc. 138, 16174−16181 (2016)
$RhSe_2$	1 M KOH	81.6	96	Adv. Mater. 33, 2007894 (2021)
OsP_2@NPC	1.0 M KOH	90	54	Chem. Commun. 55, 4399-4402 (2019)
Pt@PCM	1.0 M KOH	139	74	Sci. Adv. 4, eaao6657 (2018)

Supplementary Table 3. Summary of recently reported catalysts for HER in acidic electrolyte.

Catalyst	Electrolyte	Overpotential @10 mA cm^{-2}	Tafel slope /mV.dec^{-1}	Ref.
**B-Os Aerogel**	0.5 M H_2SO_4	**12**	**26.8**	**This work**
**Pt/C**	0.5 M H_2SO_4	**16**	**30.1**	**This work**
Ru@MWCNT	0.5 M H_2SO_4	13	27	Nat. Commun. 11, 1-10 (2020)
Ru@GnP	0.5 M H_2SO_4	13	30	Adv. Mater. 30, 1803676 (2018)
$NiRu_{0.13}$-BDC	1.0 M HCl	13	N/A	Nat. Commun. 12, 1-8 (2021)
Co-RuIr	0.1 M HClO4	13.8	31.1	Adv. Mater. 31, 1900510 (2019)
Rh_2P	0.5 M H_2SO_4	14	31.7	Adv. Energy Mater. 8, 1703489 (2018)
Ir-NSG	0.5 M H_2SO_4	17	19.2	Nat. Commun. 11, 1-10 (2020)
Pt_1/N-C	0.5 M H_2SO_4	19	14.2	Nat. Commun. 11, 1-8 (2020)
Pt_5/HMCS	0.5 M H_2SO_4	20.7	28.3	Adv. Mater. 32, 1901349 (2020)
Ru@C_2N	0.5 M H_2SO_4	22	30	Nat. Nanotechnol. 12, 441-446 (2017)
IrCo@NC-500	0.5 M H_2SO_4	24	23	Adv. Mater. 30, 1705324 (2018)
Au@ AuIr2	0.5 M H_2SO_4	29	15.6	J. Am. Chem. Soc. 143, 4639-4645 (2021)
Pt-SAs/WS_2	0.5 M H_2SO_4	32	28	Nat. Commun. 12, 1-11 (2021)
IrCoNi	0.1 M $HClO_4$	33	31.9	Adv. Mater. 29, 1703798 (2017)
a-$RuTe_2$ PNRs	0.5 M H_2SO_4	33	35	Nat. Commun. 10, 1-11 (2019)
Pt_1/OLC	0.5 M H_2SO_4	38	36	Nat. Energy 4, 512-518 (2019)

RuP_2@NPC	0.5 M H_2SO_4	38	38	Angew. Chem. Int. Ed. 56, 11559-11564 (2017)
OsP_2@NPC	0.5 M H_2SO_4	38	40	J. Catal. 370, 404-411 (2019)
OsP_2@NPC	0.5 M H_2SO_4	46	43	Chem. Commun. 55, 4399-4402 (2019)
RuIr-NC	0.05 M H_2SO_4	46	32	Nat. Commun. 12, 1-9 (2021)
$RhSe_2$	0.5 M H_2SO_4	49.9	39	Adv. Mater. 33, 2007894 (2021)
Ni@Ni_2P-Ru	0.5 M H_2SO_4	51	35	J. Am. Chem. Soc. 140, 2731-2734 (2018)
Li-$IrSe_2$	0.5 M H_2SO_4	55	N/A	Angew. Chem. Int. Ed. 131, 14906-14911 (2019)
Pt@PCM	1.0 M KOH	105	63.7	Sci. Adv. 4, eaao6657 (2018)
2.20wt% Ru SAs-Ni_2P	0.5 M H_2SO_4	125	71	Nano Energy 80, 105467 (2021)
Ru@CN	0.5 M H_2SO_4	126	N/A	Energy Environ. Sci. 11, 800-806 (2018)

Supplementary Table 4. Summary of recently reported catalysts for HER in neutral electrolyte.

Catalyst	Electrolyte	Overpotential @10 mA cm^{-2}	Tafel slope /mV.dec^{-1}	Ref.
**B-Os Aerogel**	**1.0 M PBS**	**33**	**44.7**	**This work**
**Pt/C**	**1.0 M PBS**	**62**	**78.1**	**This work**
RuNi/CQDs	1.0 M PBS	18	76	Angew. Chem. Int. Ed. 59, 1718-1726 (2020)
Ir-NSG	1.0 M PBS	22	21.2	Nat. Commun. 11, 1-10 (2020)
Pt SA-NT-NF	1.0 M PBS	24	30	Angew. Chem. Int. Ed. 56, 13694-13698 (2017)
$NiRu_{0.13}$-BDC	1.0 M PBS	36	32	Nat. Commun. 12, 1-8 (2021)
Rh_2P	1.0 M PBS	38	46	Adv. Energy Mater. 8, 1703489 (2018)
RhP_x@NPC	1.0 M PBS	38	56	Adv. Funct. Mater. 29, 1901790 (2019)
RuS_x/S-GO	1.0 M PBS	46	39	Small 15, 1904043 (2019)
1.08 wt% Pt/N-Mo_2C	1.0 M PBS	49	86.74	Small 15, 1900014 (2019)
OsP_2@NPC	1.0 M PBS	54	82	J. Catal. 370, 404-411 (2019)
Ru@Co-SAs/N-C	1.0 M PBS	55	82	Nano Energy 59, 472-480 (2019)
RhCu NTs	0.1 M PBS	57	95	Adv. Energy Mater. 10, 1903038 (2020)
RuP_2@NPC	1.0 M PBS	57	87	Angew. Chem. Int. Ed. 56, 11559-11564 (2017)
Ru_2B_3@BNC	1.0 M PBS	58	69.9	Nano Energy 75, 104881 (2020)
PtNi@Ti_3C_2 MXene	1.0 M PBS	67	70	Appl. Catal. B Environ. 291, 120100 (2021)
Re_3P_4@NPVC	1.0 M PBS	70	77	Appl. Catal. B Environ. 256, 117851 (2019)

Ru/OMSNNC	1.0 M PBS	70	N/A	Adv. Mater. 33, 2006965 (2021)
PdP_2@CB	1.0 M PBS	84.6	72.3	Angew.Chem. Int.Ed. 57, 14862-14867 (2018)
L-RuP	1.0 M PBS	95	54	Adv. Mater. 30, 1800047 (2018)
Ru@CN	1.0 M PBS	100	N/A	Energy Environ. Sci. 11, 800-806 (2018)
RhCoB aerogel	1.0 M PBS	113	149.1	J. Mater. Chem. A 8, 5595-5600 (2020)
Li-$IrSe_2$	1.0 M PBS	120	N/A	Angew. Chem. Int. Ed. 131, 14906-14911 (2019)
OsP_2@NPC	1.0 M PBS	144	64	Chem. Commun. 55, 4399-4402 (2019)
RhCu NWs-2	1.0 M PBS	165	211	Adv. Energy Mater. 10, 1903038 (2020)
2.20wt% Ru SAs-Ni_2P	1.0 M PBS	260	N/A	Nano Energy 80, 105467 (2021)
NiRu@NC	1.0 M PBS	482	N/A	J. Mater. Chem. A 6, 1376-1381 (2018)

Supplementary Table 5. B contents in samples synthesized using different concentration of $NaBH_4$.

**Samples**	**Os (B)-1**	**Os (B)-2**	**Os (B)-3**	**Os (B)-4**
$NaBH_4$ (mM)	5	50	500	5000
B (%)	0.48	0.67	0.73	0.85

Supplementary Table 6. Summary of as-prepared noble metal aerogels for HER in alkaline electrolyte.

Catalyst	C_{dl} / mF cm^{-2}	Overpotential @10 mA cm^{-2}	Tafel slope / mV.dec^{-1}
**B-Os Aerogel**	**42.85**	**19**	**35.8**
Pt	35.95	33	41.9
Rh	9.66	43	60.6
Ru	30.93	45	62.9
Pd	20.11	55	80.6
Ir	10.97	108	123.8
Au	0.86	582	128.7
Ag	0.58	634	172.3

Supplementary Table 7. Calculated doping formation energy, defined as $E_{form(I)} = E_{tot}(Os\text{-}B) - E_{tot}(Os) - nE(B)$ for interstitial doping and $E_{form(S)} = E_{tot}(Os\text{-}B) - E_{tot}(Os) - nE(B) + nE(Os)$ for substitute doping. $E_{tot}(Os\text{-}B)$, $E_{tot}(Os)$, E(B) and E(Os) represent the total energy of Os slab with B doping, Os slab, one B atom, one Os atom, respectively. Here, n is the number of B atoms.

Doped system	Formation energy (eV)
Os-1B(S)	0.61
Os-2B(S)	0.80
Os-3B(S)	1.26
Os-4B(S)	0.93
Os-1B(I)	–1.61
Os-2B(I)	–2.69
Os-3B(I)	–3.68
Os-4B(I)	–0.10
